# Identification of Deregulated miRNAs and mRNAs Involved in Tumorigenesis and Detection of Glioblastoma Patients Applying Next-Generation RNA Sequencing

**DOI:** 10.3390/ph18030431

**Published:** 2025-03-19

**Authors:** Dóra Géczi, Álmos Klekner, István Balogh, András Penyige, Melinda Szilágyi, József Virga, Andrea Bakó, Bálint Nagy, Bernadett Torner, Zsuzsanna Birkó

**Affiliations:** 1 Department of Human Genetics, Faculty of Medicine, University of Debrecen, H-4032 Debrecen, Hungary; g.dora@med.unideb.hu (D.G.); balogh@med.unideb.hu (I.B.); penyige@med.unideb.hu (A.P.); szilagyi.melinda@med.unideb.hu (M.S.); nagy.balint@med.unideb.hu (B.N.); torner.bernadett@med.unideb.hu (B.T.); 2 Department of Neurosurgery, Faculty of Medicine, University of Debrecen, H-4032 Debrecen, Hungary; klekner.almos@med.unideb.hu; 3 Division of Clinical Genetics, Department of Laboratory Medicine, Faculty of Medicine, University of Debrecen, H-4032 Debrecen, Hungary; 4 Department of Oncology, Faculty of Medicine, University of Debrecen, H-4032 Debrecen, Hungary; virga.jozsef@med.unideb.hu (J.V.); bako.andrea@lib.unideb.hu (A.B.)

**Keywords:** glioblastoma, miRNAs, brain tissue, next-generation sequencing, biomarker

## Abstract

(1) **Background:** Glioblastoma (GBM) is one of the most aggressive brain tumors with a poor prognosis. Therefore, new insights into GBM diagnosis and treatment are required. In addition to differentially expressed mRNAs, miRNAs may have the potential to be applied as diagnostic biomarkers. (2) **Methods:** In this study, profiling of human miRNAs in combination with mRNAs was performed on total RNA isolated from tissue samples of five control and five GBM patients, using a high-throughput RNA sequencing (RNA-Seq) approach. (3) **Results:** A total of 35 miRNAs and 365 mRNAs were upregulated, while 82 miRNAs and 1225 mRNAs showed significant downregulation between tissue samples of GBM patients compared to the control samples using the iDEP *tool* to analyze RNA-Seq data. To validate our results, the expression of five miRNAs (hsa-miR-196a-5p, hsa-miR-21-3p, hsa-miR-10b-3p, hsa-miR-383-5p, and hsa-miR-490-3p) and fourteen mRNAs (E2F2, HOXD13, VEGFA, CDC45, AURKB, HOXC10, MYBL2, FABP6, PRLHR, NEUROD6, CBLN1, HRH3, HCN1, and RELN) was determined by RT-qPCR assay. The miRNet tool was used to build miRNA–target interaction. Furthermore, a protein–protein interaction (PPI) network was created from the miRNA targets by applying the NetworkAnalyst 3.0 tool. Based on the PPI network, a functional enrichment analysis of the target proteins was also carried out. (4) **Conclusions:** We identified an miRNA panel and several deregulated mRNAs that could play an important role in tumor development and distinguish GBM patients from healthy controls with high sensitivity and specificity using total RNA isolated from tissue samples.

## 1. Introduction

Glioblastoma (GBM; World Health Organization grade 4) is the most common and aggressive primary brain cancer in adults and is considered to be the most prevalent form of brain tumor leading to death [1]. This incurable malignant tumor has a median survival time of about 15 months from diagnosis; the 5-year survival rate is only 10%. Typically, GBM appears in the sixth decade of life, and it is slightly predominant in males [2]. The current standard of treatment (called “Stupp’s regimen”) includes surgical resection of the tumor followed by radiotherapy combined with adjuvant temozolomide (TMZ) chemotherapy [3]. Additionally, personalized therapeutic agents against specific deregulated targets that could be responsible for the induction of tumor growth have already been tested in several clinical trials. However, almost all GBM patients undergo unavoidable tumor recurrence [4]. The molecular mechanisms behind the development of GBM are still not completely understood despite the recent achievements in GBM research. Therefore, it is crucially important to identify other factors that could contribute to the onset and progression of GBM. According to that goal, epigenetics and epigenetic modulators have come into focus that are involved in the development of cancer [5].

The latest members of the epigenetic machinery are the noncoding RNAs (ncRNAs), which either have a limited ability to encode a protein or lack it. MicroRNAs (miRNAs) are small 21–25 nucleotide-long ncRNAs that function as major players in the post-transcriptional regulation of protein-coding genes via their sequence-specific binding to the 3′ untranslated regions (UTR) of target mRNAs [6]. The results of different research groups show that miRNAs as post-transcriptional regulators are involved in the regulation of several important processes, such as cell differentiation, cell division, apoptosis, cell metabolism, and patterning of the nervous system [7]. Different mechanisms like defects in miRNA biogenesis, abnormalities in miRNA processing, and epigenetic alterations and mutations in the miRNA recognition sites of target genes can lead to changes in miRNA expression and, as a consequence, can initiate tumorigenesis [8,9]. In the case of various cancers, many miRNAs show tumor-specific expression patterns and significant deregulation. MiRNAs can function as either oncogenes (oncomiRs) or tumor suppressors; however, because of the large number of target genes, the same miRNA may play opposing roles in different tumor types [10]. Characterizing miRNA expression in GBM could be applied as a potential diagnostic or prognostic tool; furthermore, miRNAs and their targets may be helpful in selecting appropriate therapy [11]. Additionally, the simultaneous analysis of mRNA expression can add more details to the analysis of regulatory networks of differently expressed (DE) miRNAs in GBM.

The objective of this study was to profile miRNA expression in conjunction with mRNA expression in identical tissue samples of GBM patients (GPs), who were diagnosed and treated at the Department of Neurosurgery, Faculty of Medicine, University of Debrecen, Hungary, to detect those miRNAs and mRNAs whose significant deregulation correlates with tumorigenesis. Previously, it was shown that ethnicity seemed to be one of the potential sources of heterogeneity between studies [12,13]; therefore, it is inevitable to screen for significantly deregulated miRNAs that are specific for a patient cohort at a certain geographic location to create a miRNA panel that could be applied for diagnosis of GBM in that specific region. The limitation of the use of a single-miRNA biomarker is that its significant deregulation could be associated with various types of cancers, so it is sensible to apply a combination panel of miRNAs in cancer diagnosis [14]. In our work, we applied a high-throughput next-generation RNA sequencing method and bioinformatics analysis to determine the significantly deregulated miRNAs, together with mRNAs in the same tissue samples of GPs. The identified miRNAs and their targets were used to build interaction networks, which were subjected to Gene Ontology (GO) and pathway enrichment analyses in order to identify the significant molecular pathways that could be affected by the deregulated miRNA pattern. The identification of miRNAs whose expression was significantly different and their respective targets in the tissue samples of GPs could provide further insights and facilitate our understanding of the pathogenesis of GBM.

## 2. Results

### 2.1. Identification of Differently Expressed (DE) miRNAs in Tissue Samples of GBM Patients and Control Group

#### 2.1.1. Next-Generation Sequencing (NGS)

To analyze the alterations in miRNA expression patterns in tissue samples of GPs, next-generation sequencing (NGS) technology was employed using an Illumina NextSeq 500 instrument (Illumina, San Diego, CA, USA). Small RNA-Seq sequencing libraries were generated from total RNA prepared from the surgically removed tumor tissue of five GPs and the peripheral tumor region of five lower-grade (grade 1–2) glioma patients, serving as a control group.

#### 2.1.2. Hierarchical Clustering with Heatmap and Principal Component (PCA) Analysis

**Figure 1:**
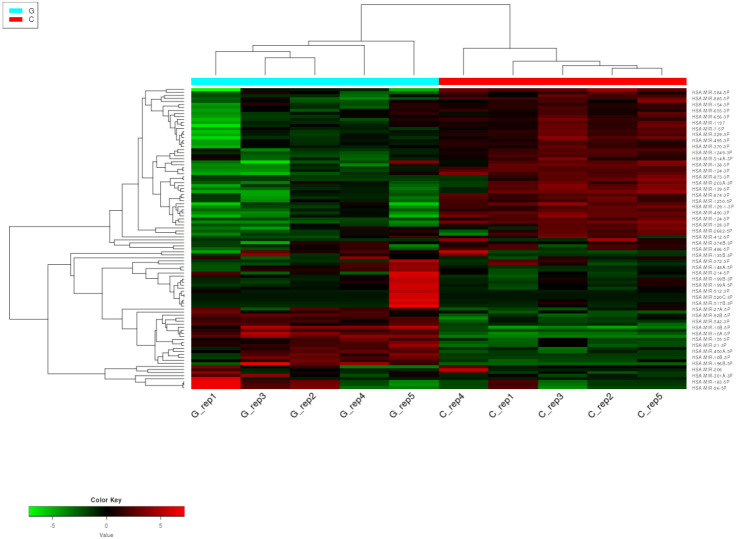
Heatmap with hierarchical clustering dendrogram of miRNA expression in GBM and control samples based on the variation in miRNA expression. The expression profiles of the top 50 miRNAs are shown. Columns represent patient and control individuals (G—glioblastoma sample and C—control sample), and each row represents a single miRNA. The down- and upregulated miRNAs are labeled green and red, respectively.

To visualize the differences in the expression patterns of miRNAs between GPs and controls, a hierarchical cluster analysis was performed on the NGS dataset of five replicates for each of the two groups to identify miRNAs with similar expression patterns. MiRNAs were ranked based on their standard deviation across all samples, and the top 200 genes were used in hierarchical clustering. Expression profiles of the top 50 most variable miRNAs in the samples of GPs and controls are depicted as a heatmap in Figure 1.

**Figure 2:**
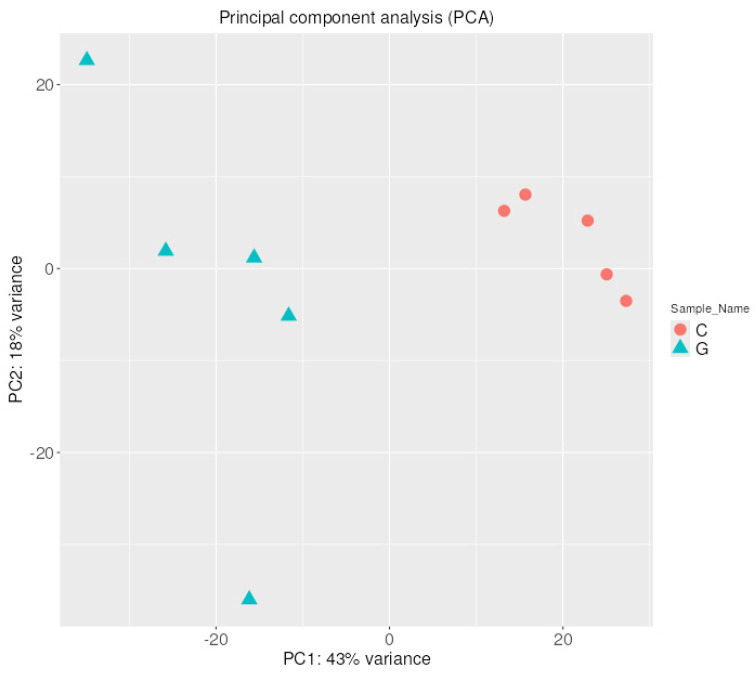
PCA of miRNA expression based on their expression profile. A clear separation is visible between the GBM samples and the control samples along the first principal component.

Principal component analysis (PCA) was also performed to increase interpretability and visualize the distribution of miRNA expression values. A PCA plot using the first and second principal components is presented in Figure 2. GBM samples form a single cluster based on their expression pattern and are clearly separated from the control samples by the first principal component, which represents 42% of the variance. This data distribution suggests that GBM biogenesis induces a drastic change in the expression of several miRNAs.

#### 2.1.3. Differentially Expressed Genes (DEGs)

Using the DESeq2 algorithm of the iDEP.96 web tool and applying thresholds of false discovery rate (FDR) < 0.05 and fold-change > 2, we identified 117 miRNAs whose expression proved to be significantly different in GPs compared to the control samples. Among the 117 differently expressed miRNAs, 35 showed upregulation (log_2_FC ≥ 1), and 82 showed downregulation (log_2_FC ≤ −1). The whole list of the 117 deregulated miRNAs is presented in Table S1. The four most strongly upregulated miRNAs were hsa-miR-10b-5p (log_2_FC = 6.9), hsa-miR-196a-5p (log_2_FC = 5.62), hsa-miR-10a-5p (log_2_FC = 5.48), and hsa-miR-21-3p (log_2_FC = 4.39). Conversely, the four most downregulated miRNAs were hsa-miR-383-5p (log_2_FC = −6.3), hsa-miR-129-5p (log_2_FC = −5.96), hsa-miR-129-2-3p (log_2_FC = −5.89), and hsa-miR-219a-2-3p (log_2_FC = −5.8).

**Figure 3:**
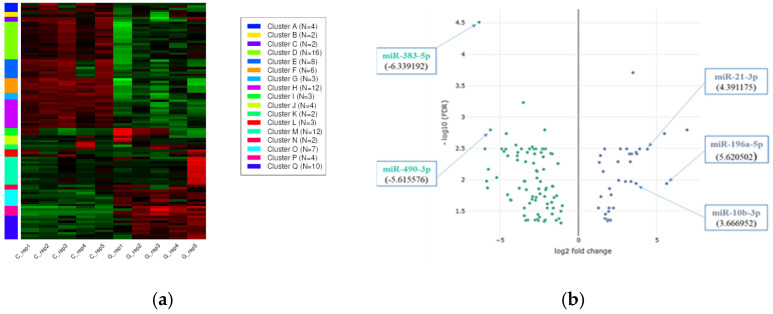
(**a**) K-means clustering of the 117 differentially expressed miRNA genes detected with the DESeq2 algorithm. The down- and upregulated miRNAs are labeled green and red, respectively. (**b**) The volcano plot shows that GBM development leads to a massive change in the miRNA transcriptome. The labeled miRNAs (hsa-miR-196a-5p, hsa-miR-21-3p, hsa-miR-10b-3p, hsa-miR-383-5p, and hsa-miR-490-3p) were included in the validation group by RT-qPCR.

To assess the precise effect of GBM development on miRNA expression patterns compared to control samples, k-means clustering was used to cluster miRNAs into groups by their expression values (Figure 3a). The heatmap and volcano plot of the expression values (Figure 3b) show that GBM development results in a massive change in the miRNA transcriptome.

#### 2.1.4. miRNA Ranking by Network-Based Analysis

**Figure 4:**
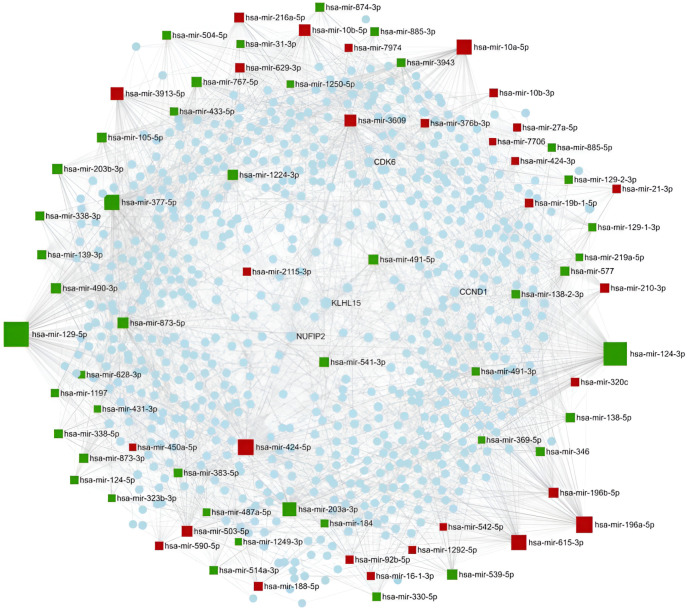
The minimum network of the differentially expressed (DE) miRNAs and their experimentally validated target genes. The network was constructed using the miRNet tool. Color code: A red square indicates an upregulated miRNA, while a green square indicates a downregulated miRNA; the blue spots are proteins.

miRNAs can function either as oncogenes or tumor suppressors; however, identifying their targets can facilitate the elucidation of their role in GBM development. Since a single miRNA regulates multiple genes, and a combination of miRNAs can co-modulate signaling pathways, the application of network-based approaches is inevitable to understand the contribution of an exact miRNA to GBM development. Our analysis was based on experimentally validated miRNA targets, applying the miRNet tool. A miRNA-centric network was constructed, including direct miRNA–target gene interactions and target gene-coded protein–protein interactions (PPI). In this heterogeneous network, hsa-miR-15a-5p has the highest degree value (260), followed by hsa-miR-424-5p (185) and hsa-miR-21-5p (131), reflecting their importance in the interactome network. Regarding the target genes, Nuclear FMR1 Interacting Protein 2 (NUFIP2) was regulated by the highest number of miRNAs and had the most interacting protein partners (degree value 20), followed by Zing Finger Protein 460 (ZNF460; degree value 19), Cyclin D1 (CCND1; degree value 15), and Cyclin-Dependent Kinase 6 (CDK6; degree value 15). The minimum network built from the miRNA–target gene and protein–protein interactions—as generated by the miRNet tool—is shown in Figure 4. The red squares represent the upregulated miRNAs, while the green squares represent the downregulated miRNAs.

#### 2.1.5. Gene Ontology (GO) and Pathway Enrichment Analysis of miRNA Targets

**Figure 5:**
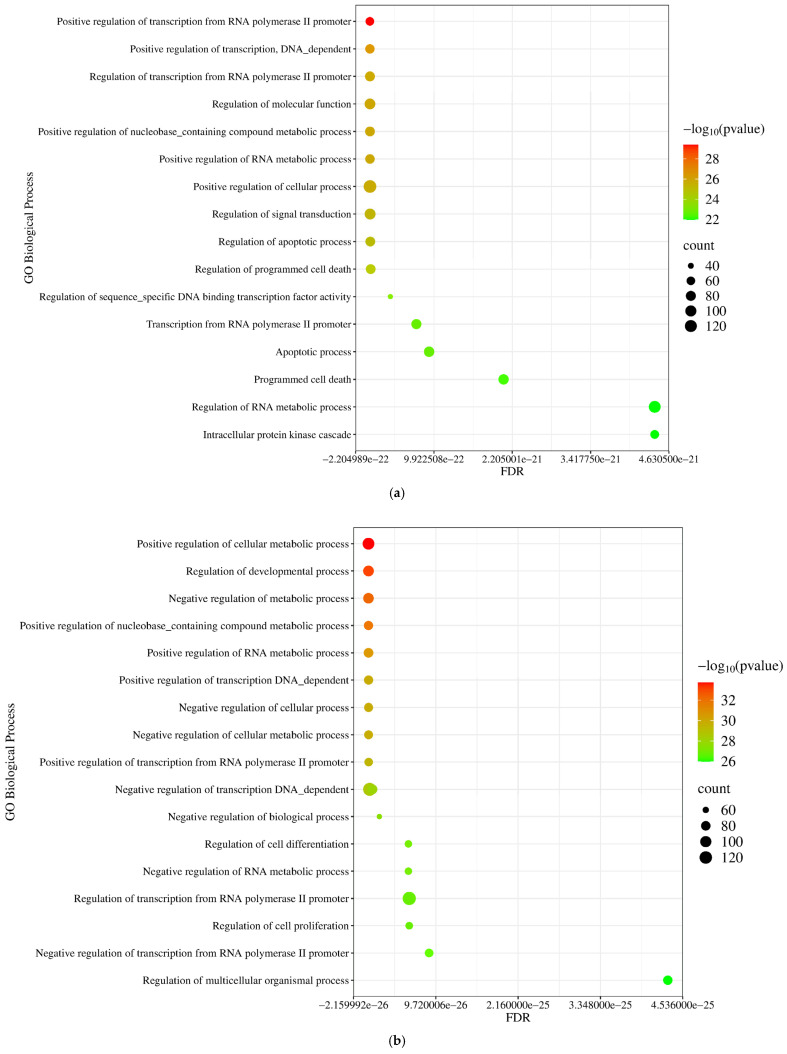
GO Biological Process-based functional enrichment annotation of (**a**) upregulated and (**b**) downregulated miRNA-related target genes using the NetworkAnalyst 3.0 tool. The significance of the detected biological processes is characterized by their FDR and −log_10_ *p*-values. The size of the dots is proportional to the number of genes included in the given process.

Using the GO and Kyoto Encyclopedia of Genes and Genomes (KEGG) database options of the NetworkAnalyst 3.0 tool, functional enrichment and pathway analysis were performed based on the PPIs of experimentally validated miRNA targets. As shown in Figure 5a, the protein products of the target genes of the upregulated miRNAs were enriched in biological processes such as positive regulation of the metabolic process (*p* = 2.11 × 10^−30^), regulation of signal transduction (*p* = 4.54 × 10^−26^), and regulation of apoptotic process (*p* = 9.54 × 10^−26^). In contrast, the proteins of the downregulated miRNAs were involved in the regulation of developmental processes (*p* = 1.37 × 10^−33^), negative regulation of metabolic processes (*p* = 5.21 × 10^−33^), and positive regulation of transcription (*p* = 1.4 × 10^−30^) (Figure 5b).

**Figure 6:**
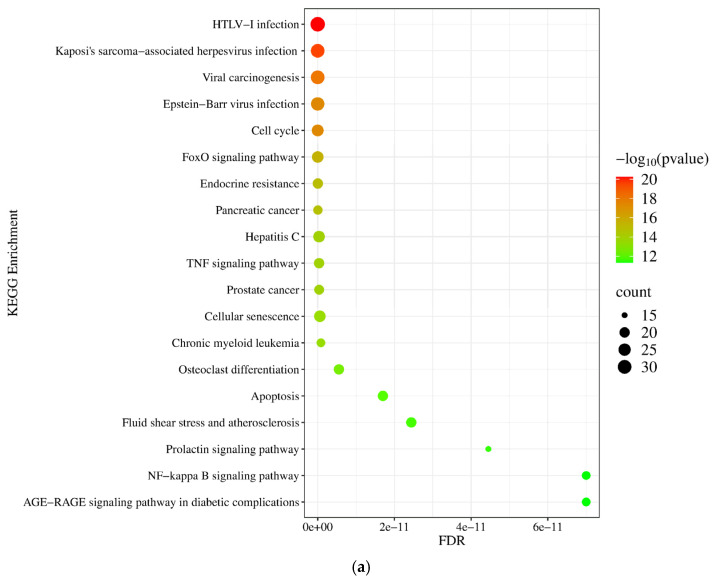

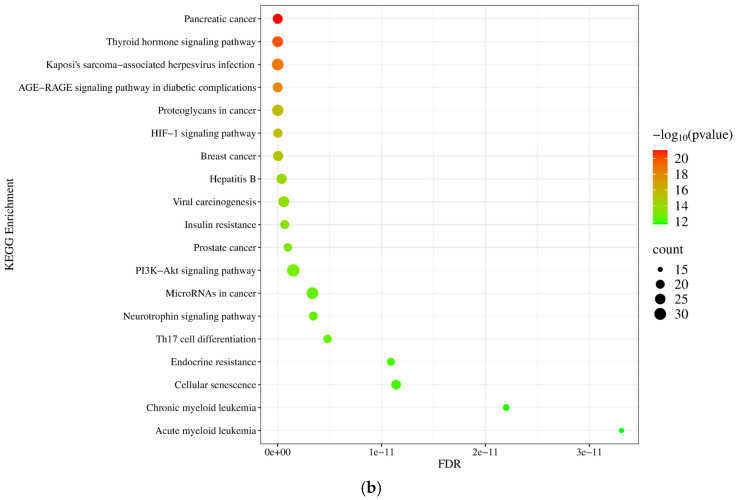
The KEGG pathway-based functional pathway enrichment analysis of target genes (**a**) of upregulated and (**b**) downregulated miRNAs using the NetworkAnalyst 3.0 tool. The significance of the detected KEGG pathways is specified by their FDR and −log10 *p*-values. The size of the dot reflects the number of genes included in the given pathway.

According to the KEGG database, we have found that the target genes of upregulated miRNAs are implicated in diverse cancer-related pathways, including the pathways in cancer (*p* = 1.22 × 10^−21^), cell cycle (*p* = 5.33 × 10^−189^), and FoxO signaling pathway (*p* = 3.22 × 10^−16^) (Figure 6a), while the downregulated miRNAs are involved in the regulation of the AGE-RAGE signaling pathway (*p* = 1.3 × 10^−18^) and proteoglycan in cancer (*p* = 2.25 × 10^−16^) (Figure 6b).

#### 2.1.6. Validation of Differentially Expressed (DE) miRNAs by RT-qPCR in Tissue Samples

**Figure 7:**
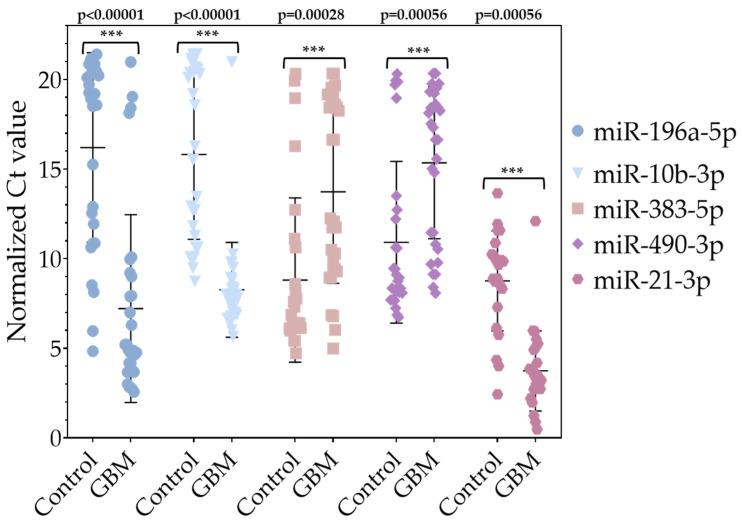
Representation of normalized Ct values of significantly deregulated miRNAs. The *p*-values were calculated using the Mann–Whitney U test. *** *p* < 0.001. The *p*-value of all validated miRNAs is *p* < 0.01.

To validate the results obtained by NGS, three upregulated miRNAs (hsa-miR-196a-5p (log_2_FC = 5.6), hsa-miR-21-3p (log_2_FC = 4.39), and hsa-miR-10b-3p (log_2_FC = 3.66)) and two downregulated miRNAs (hsa-miR-383-5p (log_2_FC = −6.33) and hsa-miR-490-3p (log_2_FC = −5.61)) were selected for an RT-qPCR analysis. For validation, total RNA was isolated from tissue samples from 30 GPs and 28 control samples from the peripheral tumor region of patients with low-grade (grade 1–2) glioma, which served as a control group. The number of women and men was equal in both groups. RT-qPCR was used to measure the relative expression of these miRNAs using hsa-miR-103a-3p as a reference miRNA [15]. All measurements were performed in triplicate. According to the results of the RT-qPCR reactions using the Mann–Whitney U test for calculation, we confirmed that the expression of hsa-miR-196a-5p, hsa-miR-21-3p, and hsa-miR-10b-3p was significantly upregulated, while hsa-miR-383-5p and hsa-miR-490-3p showed significant downregulation compared to their expression in the control samples (Figure 7).

**Figure 8:**
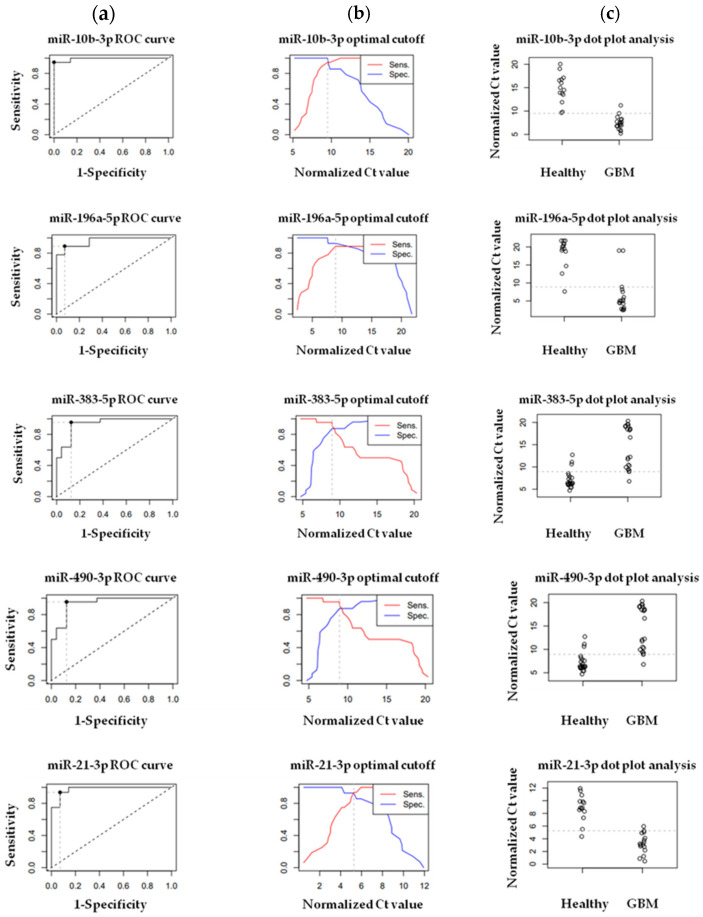
(**a**) ROC (Receiver Operating Characteristics) curves with AUC (area under the curve) were created to detect and represent the sensitivity and specificity of hsa-miR-10b-3p, hsa-miR-196a-5p, hsa-miR-383-5p, hsa-miR-490-3p, and hsa-miR-21-3p selected for the validation of the NGS via RT-qPCR. (**b**) Calculated optimal cut-off point values of hsa-miR-10b-3p (normalized Ct value: 9.9), hsa-miR-196a-5p (normalized Ct value: 9.8), hsa-miR-383-5p (normalized Ct value: 9.8), hsa-miR-490-3p (normalized Ct value: 9.8), and hsa-miR-21-3p (normalized Ct value: 5.8). (**c**) Dot plot analysis of hsa-miR-10b-3p, hsa-miR-196a-5p, hsa-miR-383-5p, hsa-miR-490-3p, and hsa-miR-21-3p for GPs and control tissue samples.

We created ROC-AUC curves applying the normalized expression data resulting from the RT-qPCR measurements of hsa-miR-196a-5p, hsa-miR-21-3p, hsa-miR-10b-3p, hsa-miR-383-5p, and hsa-miR-490-3p in order to verify their diagnostic potential. The ROC-AUC values were 0.96032, 0.97768, 0.99206, 0.9375, and 0.9648 in the case of hsa-miR-196a-5p, hsa-miR-21-3p, hsa-miR-10b-3p, hsa-miR-383-5p, and hsa-miR-490-3p, respectively. For the control group and GPs, the normalized Ct values were dichotomized by mapping the sensitivity values in relation to 1—specificity in the case of hsa-miR-196a-5p, hsa-miR-21-3p, hsa-miR-10b-3p, hsa-miR-383-5p, and hsa-miR-490-3p—to calculate optimal cut-off values. We observed the highest sensitivity value in the case of hsa-miR-383-5p (95%) and hsa-miR-490-3p (95%), a little bit lower value for hsa-miR-10b-3p (94%) and hsa-miR-21-3p (93.8%), while the lowest one is in the case of hsa-miR-196a-5p (88%). The sequence of specificity was different; the highest value belonged to hsa-miR-10b-3p (100%), followed by hsa-miR-21-3p (92.9%), hsa-miR-196a-5p (92%), hsa-miR-383-5p (95%), and hsa-miR-490-3p (85%) (Figure 8).

### 2.2. Identification of Differently Expressed (DE) mRNAs in Tissue Samples of GBM Patients and Control Group

#### 2.2.1. Next-Generation Sequencing (NGS)

To assess the dissimilarities in mRNA expression profiles between the tissue samples of GPs and control individuals, next-generation sequencing (NGS) was conducted using the Illumina NextSeq 500 instrument (Illumina, San Diego, CA, USA). The RNA-Seq libraries were created from the same total RNA samples, which were isolated from resected tumor tissue of five GPs and the peripheral tumor region of five lower-grade (grade 1–2) glioma patients. These later samples were used as a control group.

#### 2.2.2. Hierarchical Clustering with Heatmap and Principal Component (PCA) Analysis

**Figure 9:**
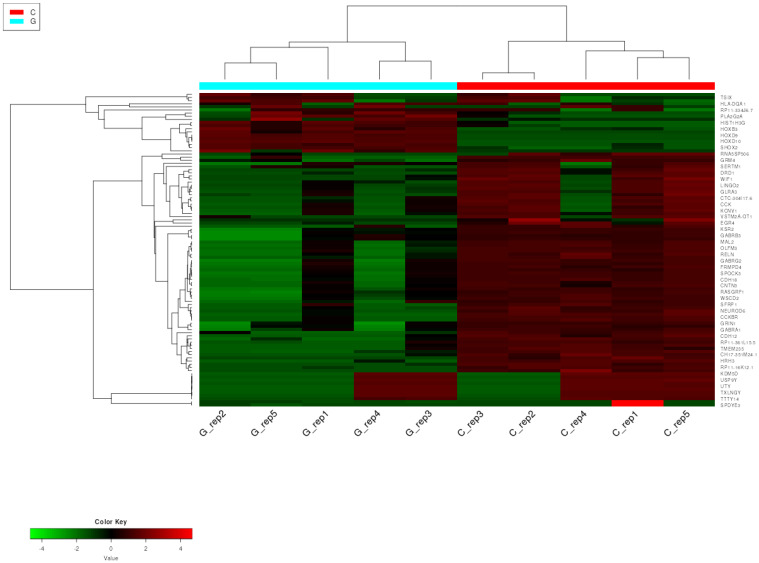
Heatmap with hierarchical clustering dendrogram of mRNA expression in GBM and control samples based on the variation in mRNA expression. The expression profiles of the top 50 mRNAs are shown. Columns represent patient and control individuals (G—glioblastoma sample and C—control sample), and each row represents a single mRNA. The down- and upregulated mRNAs are labeled green and red, respectively.

In order to interpret the results of mRNA NGS, a hierarchical cluster analysis was performed on the NGS dataset of GPs and the control group. MRNAs were ranked based on their standard deviation across all samples, and the top 200 genes were used in hierarchical clustering. The expression profiles of the top 50 most variable mRNAs in samples of GPs and controls are depicted as a heatmap in Figure 9.

**Figure 10:**
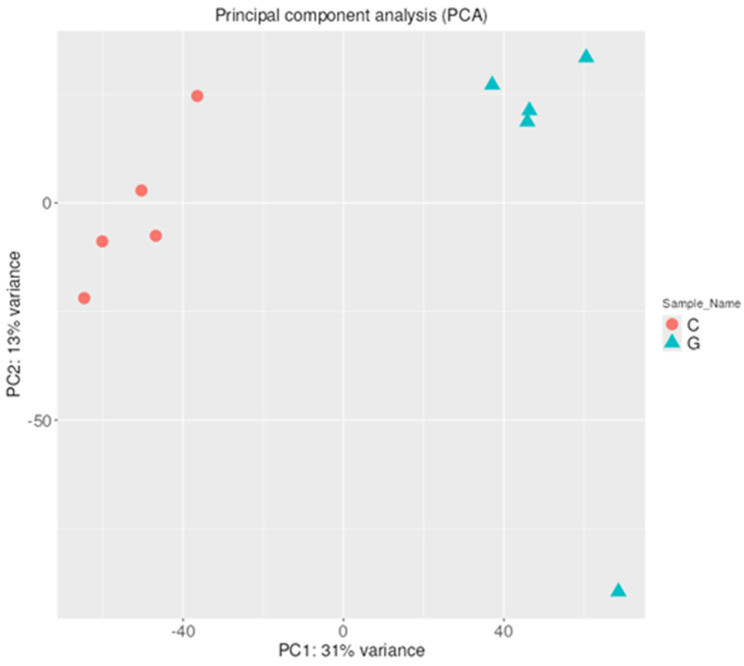
PCA of mRNA expression based on their expression profile. A clear separation is visible between the GBM samples and the control samples along the first principal component.

The distribution of mRNA expression values was visualized by Principal Component Analysis (PCA). A PCA plot using the first and second principal components is presented in Figure 10. It is visible that based on their mRNA expression pattern, GBM samples form a single cluster and are clearly separated from the control samples by the first principal component, which represents 43%. This data distribution of the variance suggests that GBM biogenesis induces a drastic change in the expression of several mRNAs.

#### 2.2.3. Differentially Expressed Genes (DEGs)

According to the DESeq2 algorithm of the iDEP.96 web tool with an adjusted threshold of false discovery rate (FDR) < 0.05 and fold-change > 2, we detected 365 upregulated (log_2_FC ≥ 1) and 1225 downregulated (log_2_FC ≤ −1) mRNAs in the GPs compared to the control samples. The whole list of the deregulated mRNAs is presented in Table S2. The five most strongly upregulated mRNAs were HOXD10 (log_2_FC = 7.06), SHOX2 (log_2_FC = 6.86), POSTN (log_2_FC = 6.09), TOP2A (log_2_FC = 6.08), and HOXD11 (log_2_FC = 5.95). In contrast, the five most significantly downregulated mRNAs were RELN (log_2_FC = −8.56), GRIN1 (log_2_FC = −8.29), UNC13C (log_2_FC = −8.04), OPALIN (log_2_FC = −7.94), and GABRA1 (log_2_FC = −7.89).

**Figure 11:**
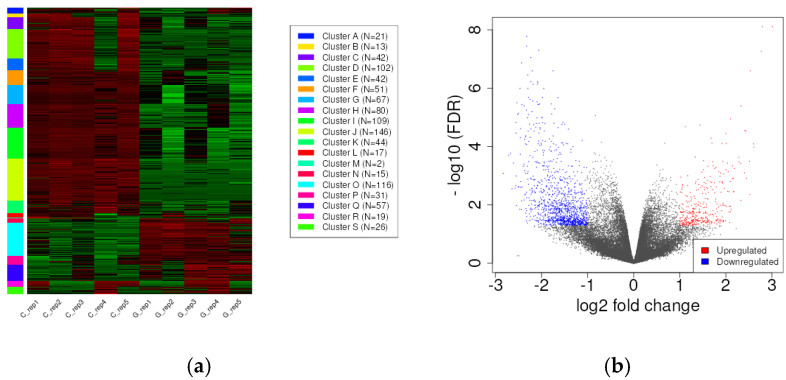
(**a**) The k-means clustering of differentially expressed (DE) mRNA genes was conducted using the DESeq2 algorithm. The down- and upregulated mRNAs are labeled green and red, respectively. (**b**) The volcano plot shows that GBM development leads to a massive change in the mRNA transcriptome. The down- and upregulated mRNAs are labeled blue and red, respectively.

To assess the influence of GBM on mRNA expression in comparison to control samples, a k-means cluster analysis was conducted to categorize mRNAs based on their expression values (Figure 11a). The volcano plot of the expression values (Figure 11b) demonstrates that GBM development results in a profound alteration of the mRNA transcriptome.

#### 2.2.4. Protein–Protein Interaction (PPI) Network Analysis of Deregulated mRNAs

**Figure 12:**
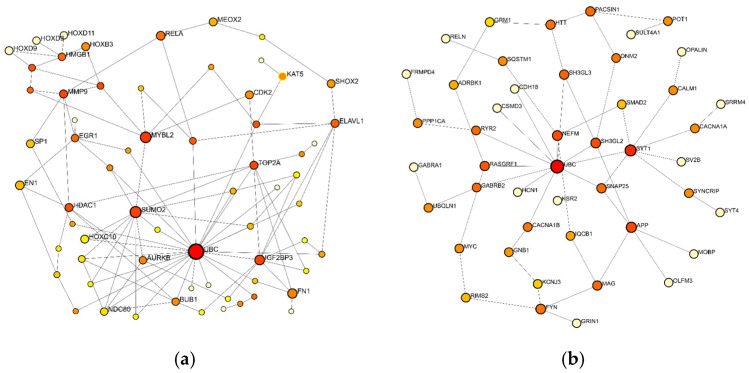
Topology of the (**a**) 50 most upregulated mRNA-based and (**b**) 50 most downregulated mRNA-based PPI minimal networks using the NetworkAnalyst 3.0 tool. Nodes indicate proteins; the size of the nodes corresponds to their degree centrality.

The construction of minimal networks based on the 50 most significantly upregulated and downregulated mRNAs was carried out using the NetworkAnalyst 3.0 tool (Figure 12). Many of the major hubs—considered to be key nodes with significant biological relevance—are proteins already known to be involved in tumorigenesis. It is an intriguing finding that the protein with the highest number of interactions in both analyses (degree values of 25 and 13 for the up- and downregulated networks, respectively) is ubiquitin C (UBC). Ubiquitination, depending on the residues involved in conjugation, can be related to the degradation of proteins, DNA repair, kinase modification, endocytosis, and regulation of the cell cycle and cell signaling pathways [16,17]. As shown in Figure 12a, following UBC, the small ubiquitin-like modifier 2 (SUMO2) and Insulin-Like Growth Factor 2 mRNA Binding Protein 3 (IGF2BP3) have relatively high degree values in the mRNA-based upregulated protein–protein interaction network (degree values of 12 and 9, respectively), while synaptotagmin-1 (SYT1, degree value 7) and Amyloid Beta Precursor Protein (APP, degree value 5) follow UBC in the downregulated mRNA-based protein–protein interaction network (Figure 12b).

#### 2.2.5. Gene Ontology (GO) and Pathway Enrichment Analysis of mRNA Molecules

**Figure 13:**
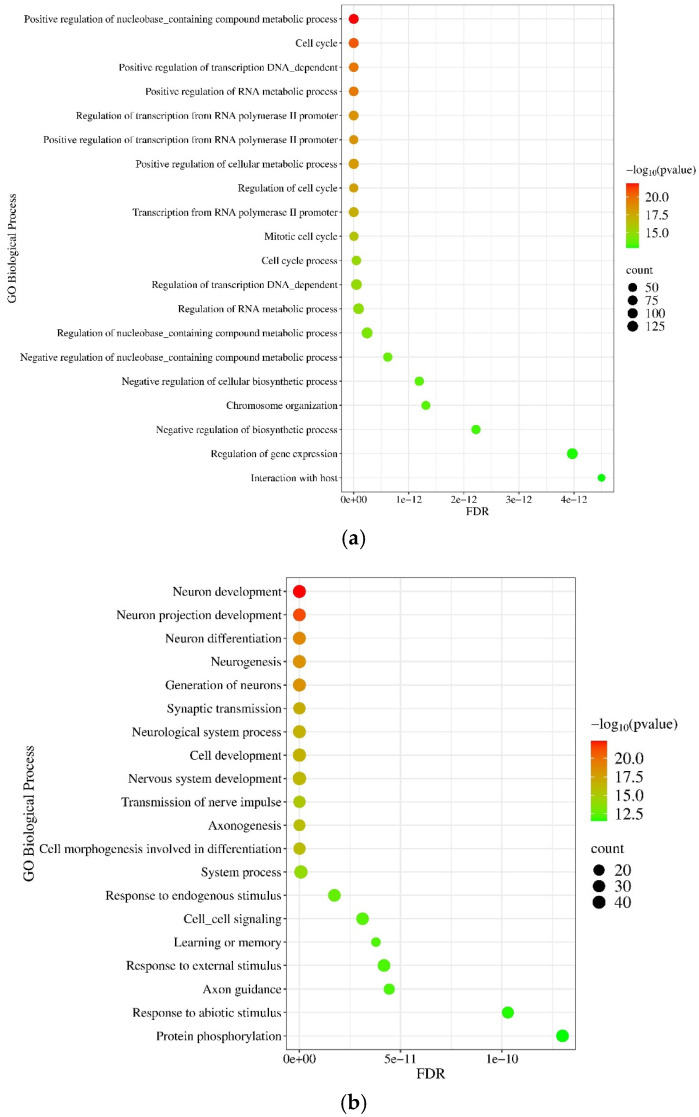
GO Biological Process-based functional enrichment annotation of (**a**) 50 most upregulated and (**b**) 50 most downregulated mRNAs using the NetworkAnalyst 3.0 tool. The significance of the detected biological processes is indicated by their FDR and −log10 *p*-values. The size of the dots is proportional to the number of genes included in the given process.

Applying the GO and Kyoto Encyclopedia of Genes and Genomes (KEGG) database options of the NetworkAnalyst 3.0 tool, PPI functional enrichment and pathway analysis were performed using deregulated mRNAs. The GO Biological Process enrichment analysis demonstrates that the upregulated proteins were implicated in the positive regulation of nucleobase-containing compound metabolic processes (*p* = 1.27 × 10^−22^), cell cycle (*p* = 2.28 × 10^−21^), positive regulation of RNA metabolic processes (*p* = 2.66 × 10^−20^), and chromosome organization (*p* = 3.35 × 10^−14^) (see Figure 13a). In contrast, the downregulated proteins were enriched in processes such as neuron development (*p* = 4.5 × 10^−23^), neuron projection development (*p* = 5.48 × 10^−22^), neuron differentiation (*p* = 1.3 × 10^−19^), and synaptic transmission (*p* = 1.43 × 10^−17^) (Figure 13b).

**Figure 14:**
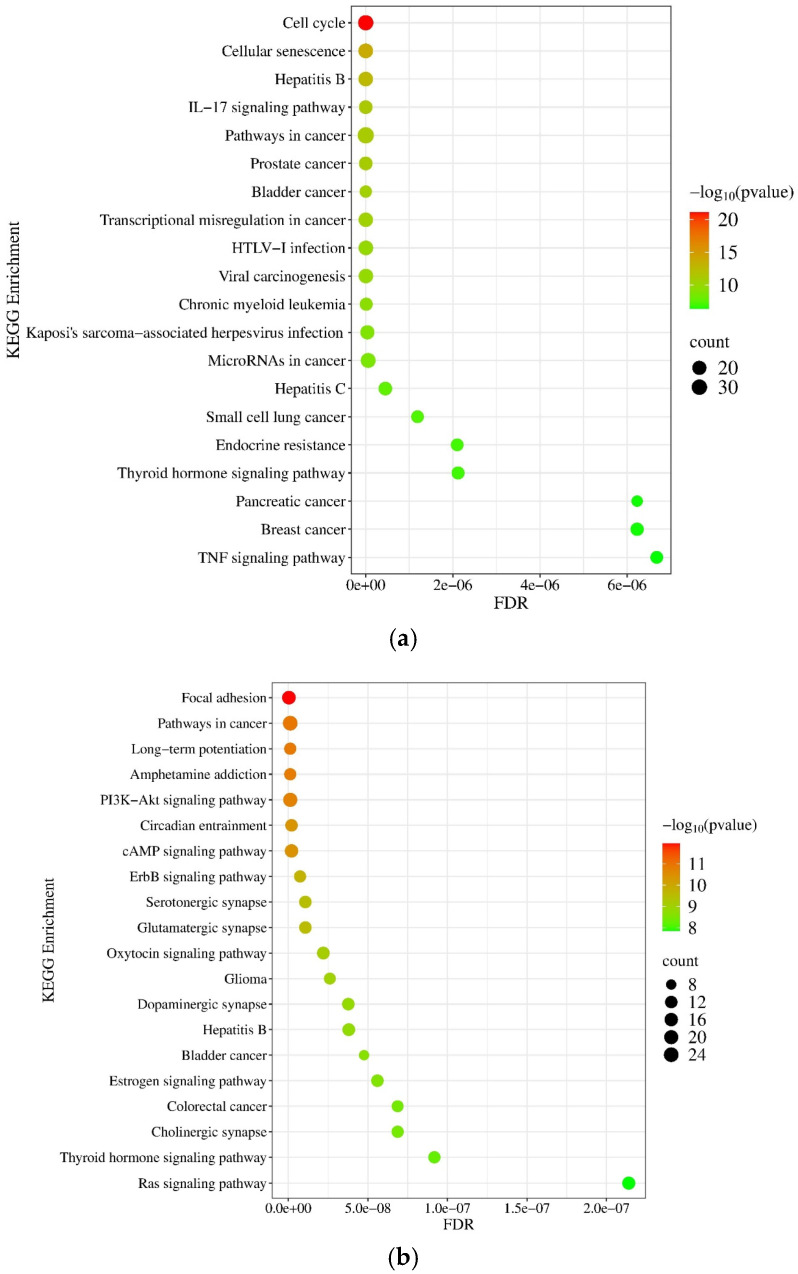
The KEGG pathway-based enrichment annotation of (**a**) 50 most upregulated and (**b**) 50 most downregulated mRNA-based analyses using the NetworkAnalyst 3.0 tool. The significance of the detected KEGG pathways is specified by their FDR and −log10 *p*-values. The size of the dot reflects the number of genes included in the given pathway.

According to the KEGG database, we found that the upregulated mRNAs were involved in different cancer-related pathways, like cell cycle (*p* = 8.31 × 10^−22^), cellular senescence (*p* = 1.69 × 10^−14^), pathways in cancer (*p* = 8.33 × 10^−12^), transcriptional misregulation in cancer (*p* = 3.06 × 10^−11^), and the IL−17 signaling pathway (*p* = 5.38 × 10^−12^) (Figure 14a), while focal adhesion (*p* = 1.13 × 10^−12^), pathways in cancer (*p* = 1.23 × 10^−11^), long-term potentiation (*p* = 1.27 × 10^−11^), and the ErbB signaling pathway (*p* = 1.85 × 10^−10^) showed downregulation (Figure 14b). It is also important to note that DEGs were highly enriched in pathways that are involved in the development of several cancer types (Figure 14).

#### 2.2.6. Validation of Differentially Expressed (DE) mRNAs by RT-qPCR in Tissue Samples

**Figure 15:**
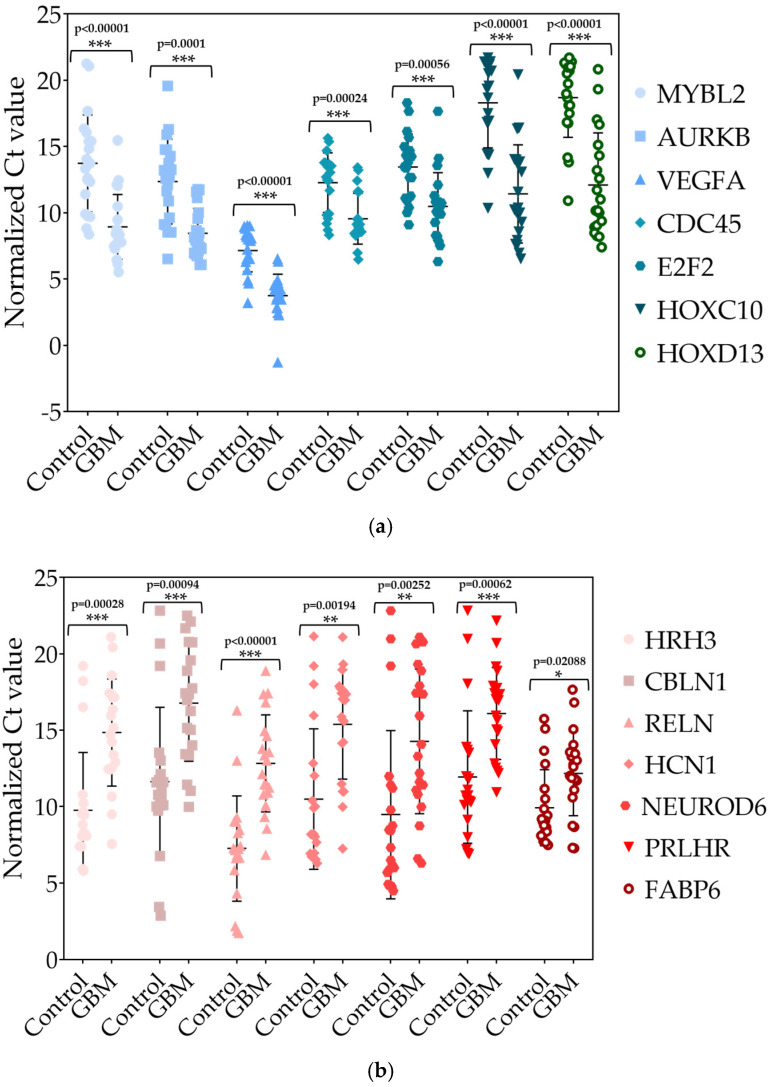
Representation of normalized Ct values of (**a**) significantly upregulated and (**b**) significantly downregulated mRNAs. The *p*-values were calculated using the Mann–Whitney U test. * *p* < 0.05; ** *p* < 0.01; *** *p* < 0.001. In the case of the upregulated mRNAs, *p* < 0.001. Related to the downregulated mRNAs, *p* < 0.001 is characteristic for HRH3, CBLN1, RELN, and PRLHR, while *p* < 0.01 is calculated in case of HCN1 and NEUROD6 and *p* < 0.05 in case of FABP6.

The same total RNA samples from GPs and the control group were used to validate the mRNA NGS results. Seven upregulated mRNAs (E2F2 (log_2_FC = 3.59), HOXD13 (log_2_FC = 3.69), VEGFA (log_2_FC = 4.3), CDC45 (log_2_FC = 4.31), AURKB (log_2_FC = 4.6), HOXC10 (log_2_FC = 4.9), and MYBL2 (log_2_FC = 5.73)) and seven downregulated mRNAs (FABP6 (log_2_FC = −2.3), PRLHR (log_2_FC = −4.37), NEUROD6 (log_2_FC = −5.72), CBLN1 (log_2_FC = −6.16), HRH3 (log_2_FC = −6.39), HCN1 (log_2_FC = −7.36), and RELN (log_2_FC = −8.5)) were chosen to check their expression in GPs by RT-qPCR. GAPDH was used as an internal control for normalizing the results [18]. In the case of the selection of deregulated mRNAs for the validation procedure, we primarily aimed to cover the entire significantly up- and downregulated expression spectrum. The list of primer sequences is presented in Table S3. Each experiment was performed in triplicate. Based on the results of RT-qPCR validation using the Mann–Whitney U test, the upregulation of E2F2, HOXD13, VEGFA, CDC45, AURKB, HOXC10, and MYBL2 (Figure 15a) and the downregulation of FABP6, PRLHR, NEUROD6, CBLN1, HRH3, HCN1, and RELN were confirmed (Figure 15b).

### 2.3. The Correlation Between miRNA and mRNA Expression Determined by Next-Generation Sequencing (NGS)

Finally, we analyzed miRNA–mRNA interactions using our miRNA and mRNA expression datasets. miRNA targets were identified using the miRTarBase and miRTargetLink 2.0 databases. Note that only experimentally validated miRNA target gene interactions were included in our study. Table 1 shows the inverse correlation between the expression of miRNAs and their experimentally validated target mRNAs in our NGS dataset. We hypothesize that miRNAs that show significant up- or downregulation in GPs may regulate the expression of genes that are involved in the cell cycle (AURKB, CDC45, and CDK6), cell proliferation (EGFR and VEGFA), and angiogenesis (VEGFA) that support tumor growth. In this context, upregulated transcription factors, such as E2F transcription factor 2 (E2F2) and MYB proto-oncogene-like 2 (MYBL2), regulate the transcription of genes involved in the cell cycle, cell differentiation, and cell proliferation. Other genes (AJAP1, MMP9, POSTN, and STC2) promote metastasis formation by regulating adhesion or migration. In addition, the upregulated Homeobox C10 (HOXC10) is involved in the transcription of genes that enhance migration capacity. The tumor microenvironment may also be influenced by the regulation of tumor-associated macrophages (through LTBP-1 and POSTN).

## 3. Discussion

GBM, which represents more than 50% of high-grade gliomas, is one of the greatest challenges facing neuro-oncology today [42]. Most GBMs arise de novo (they can develop rapidly within several weeks or months), while some GBM tumors typically develop from lower-grade gliomas [1,2]. Despite what we know about the pathogenesis of GBM, it is still incurable, and the available treatment options are still leading to short survival times. Presently, the median survival time is only 15 months following treatment, and only 10% of GPs have a 5-year survival rate [43,44]. The molecular background of GBM is still not fully understood, so there is a need to discover new molecules behind the development and progression of GBM that could also aid in diagnosis [45]. In this study, our aim was to identify key genes and miRNAs that might be involved in GBM development and can serve as potential biomarkers in the detection process.

### 3.1. Identification of Differently Expressed (DE) miRNAs and mRNAs in Tissue Samples of GBM Patients in Combination with Pathway and Gene Ontology (GO) Enrichment Analysis

Functional enrichment analysis of differently expressed (DE) miRNAs and mRNAs reveals several processes related to tumor progression (Figure 5 and Figure 13). For example, different expressions of classical neurotransmitter receptors (NTRs), like gamma-aminobutyric acid (GABA) receptors, glutamate receptors, and dopamine receptors, could contribute to tumor development. Belotti et al. detected the downregulation of 10 NTR genes in the tumor tissue samples of GPs [46]. Similarly, 5 out of 10 NTR genes (GABRA1, GABRB2, GABRG2, GRIN1, and DRD1) showed significantly decreased expression in GPs in our study. Furthermore, D’Urso et al. demonstrated that GABRA1 is post-transcriptionally regulated by hsa-miR-155 [30] (Table 1). According to the KEGG database, deregulated miRNAs might be involved in the development of several cancer types and are enriched in several cancer-related pathways, involving the FoxO signaling pathway in the case of upregulated miRNAs, while proteoglycans in cancer are related to downregulated miRNAs (Figure 6). Forkhead box O transcription factors (FOXOs), which act as tumor suppressors, play an important role, for example, in regulating cancer metabolism and angiogenesis [47]. Proteoglycans control several oncogenic pathways in cancer cells and trigger interactions between the tumor and its microenvironment. Consequently, proteoglycans, together with their modifying enzymes, could be important therapeutic targets and biomarkers of GBM [48]. Focusing on the upregulated mRNAs related pathways, upon IL-17 signaling acting via Act1-induced K63-linked ubiquitylation of TNF Receptor Associated Factor 6 (TRAF6) leads to the activation of mitogen-activated protein kinase (MAPK), CCAAT-enhancer-binding protein β (C/EBPβ), and nuclear factor κB (NF-κB) pathways [49]. Most of the downregulated mRNAs are involved in focal adhesion-associated pathways (Figure 14). The decreased expression of adhesion molecules is required for tumor cell migration and invasion [50].

### 3.2. Validation of Differentially Expressed (DE) miRNAs by RT-qPCR in Tissue Samples

Similar to our results, Takkar et al. found hsa-miR-196a to be significantly upregulated in GPs; in addition, they identified hsa-miR-196a as a hypoxia-inducible and hypoxia-inducible factor (HIF)-regulated miRNA that functions as an oncomiR in GBM [26]. Yang et al. confirmed the upregulation of hsa-miR-196a in GBM as well, and they detected a significant correlation with poor outcomes in a large cohort of GBM patients [51].

One interesting fact about hsa-miR-10b, which we found to be highly upregulated, is that it is normally undetectable in the brain but shows strong upregulation in all GBM subtypes [52]. Gabriely et al. demonstrated that hsa-miR-10b is an important oncomiR and promotes the process of tumorigenesis and maintains malignancy in GBM cells [53]. El Fatimy et al. investigated the consequences of hsa-miR-10b gene editing on GBM, and they found that its loss-of-function mutations lead to the death of glioma cells but not in other cancer cell lines. These findings suggest that virus-mediated hsa-miR-10b gene ablation could be an efficient therapeutic method in GBM treatment [54]. Hsa-miR-196a and hsa-miR-10b are situated within HOX clusters. It is noteworthy that numerous Hox miRNAs have been identified as targeting HOX mRNAs [55].

Many reports indicate the upregulation of hsa-miR-21 in GBM [56]. Cellular pathways, like p53 and the PI3K-Akt pathway, are parts of the regulatory network of hsa-miR-21 [57,58]. So, its function in tumor development and progression processes could explain the association between the upregulation of hsa-miR-21 expression and the short survival times of GPs [59].

Focusing on our downregulated miRNAs, other research groups detected significantly lower expression of hsa-miR-383-3p [60] and hsa-miR-490-3p [27] in human glioma tissues and cells as well. He et al. reported insulin-like growth factor-1 receptor (IGF1R) as a direct target of hsa-miR-383-3p, so its low expression causes the upregulation of IGF1R, leading to stimulation of AKT Serine/Threonine Kinase (AKT), which, via enhancing Matrix Metalloproteinase 2 (MMP2) production, results in increased invasion [61]. In light of the above findings, a significantly increased level of MMP9 (log_2_FC = 5.36), MMP14 (log_2_FC = 3.74), and MMP23 (log_2_FC = 2.4) could be detected on the basis of the mRNA NGS results.

Concerning hsa-miR-490-3p, Vinchure et al. identified it as an important direct target of one of the histone methylating molecules, Enhancer of Zeste Homolog-2 (EZH2). EZH2 upregulation and hsa-miR-490-3p downregulation could be an early step in tumorigenesis [41]. Similarly, both the miRNA and mRNA NGS results show the downregulation of hsa-miR-490-3p and upregulation of EZH2 (log_2_FC = 2.72).

To the best of our knowledge, the significant joint deregulation of the expression of the five miRNAs hsa-miR-196a-5p, hsa-miR-21-3p, hsa-miR-10b-3p, hsa-miR-383-5p, and hsa-miR-490-3p was only detected in our study.

One potential explanation for this finding is that ethnicity could be one of the potential sources of heterogeneity between microRNA studies [12,13]. Therefore, it may be advisable to screen for significantly deregulated microRNAs associated with a particular phenotype, such as glioblastoma multiforme (GBM), that are specific to a certain geographic area. This could result in the creation of a panel of miRNAs that could be applied for diagnostic purposes in GBM in that specific region. For example, Zhou et al., using a meta-analytical approach, found that in Caucasian populations, miRNAs displayed higher diagnostic value than in Asians, implying that deregulation of the same miRNA in different ethnicities may also influence the diagnostic value of miRNAs [62]. In addition to ethnicity, our data from the examined patient population, consistent with other GBM studies, confirm that the incidence of this disease increases with age (on average between 65–75 years) and is more frequent in males and associated with poorer median survival in males than females [63].

### 3.3. Validation of Differentially Expressed (DE) mRNAs by RT-qPCR in Tissue Samples

In the present study, the differential expression of 15 of the 39 known Hox genes was observed in GPs (see Table S2). With the exception of HOXD1 and HOXD-AS1, the HOX genes are not expressed in normal brain tissue. Consequently, their deregulated expression may play a role in the development of GBM. For instance, it has been demonstrated that the expression of HOXC10 enhances the migration capacity of GBM cell lines [64]. Furthermore, MYB Proto-Oncogene Like 2 (MYBL2), a member of the myeloblastosis transcription factor family, similar to E2F Transcription Factor 2 (E2F2), plays an important role in regulating cell proliferation and differentiation [20,65]. In addition to the transcription factors that were found to be overexpressed, Neuronal Differentiation 6 (NEUROD6) exhibited a tumor-suppressive role in GPs via the inhibition of cell proliferation, migration, and tumor formation ability in GBM cell lines. The downregulation of NEUROD6 was found to be correlated with the poor prognosis of GBM patients [22]. In addition to the previously characterized transcription factors, the validation group included other upregulated genes that are associated with the regulation of cell proliferation. For example, the cell division cycle (Cdc 45) protein plays an important role in the regulation of the initiation and elongation stages of eukaryotic chromosomal DNA replication [35]. Aurora Kinase B (AURKB), functioning as a serine/threonine kinase, plays an important role in cell cycle regulation via controlling chromatid separation. In the case of GBM, overexpression of AURKB was associated with poor prognosis and increased TMZ resistance [66]. Furthermore, Vascular Endothelial Growth Factor A (VEGFA) plays a crucial role in the induction of cell proliferation and angiogenesis, facilitating the transport of nutrients essential for tumor growth [38]. The results of the functional enrichment analysis indicate that the downregulated mRNAs are predominantly associated with neuron development and synaptic transmission (Figure 13). According to our data, the most significantly downregulated mRNA in GPs is Reelin (RELN), a large secreted extracellular matrix glycoprotein that plays an important role in brain development. It regulates the migration of neurons and the formation of layers in the cortex [67]. Neuronal excitability is regulated by hyperpolarization-activated cyclic nucleotide-gated channel 1 (HCN1), which facilitates the movement of Na^+^ and K^+^ ions across cellular membranes. There is a notable reduction in the expression of HCN1 in GBM [68]. Histamine Receptor H3 (HRH3) is normally highly expressed in the basal ganglia, cortex, and hippocampus, playing an important role in the regulation of neurotransmitter release. In contrast to the findings of Lin et al., our study shows a reduction in the expression of HRH3 [69]. Cerebellin-1 (CBLN1) is known for its role in synaptic organization and demonstrated reduced expression in line with the observations made by Nong et al. [70]. For instance, in the presynaptic region, it is capable of stimulating the accumulation of synaptic vesicles by binding with Neurexin 1 (NRXN1) [70]. Fatty acid-binding protein 6 (FABP6) and prolactin receptor (PRLHR) showed downregulation in our GPS in contrast to the findings of other research groups [71,72].

### 3.4. The Correlation Between miRNA and mRNA Expression Determined by Next-Generation Sequencing (NGS)

The miRNAs listed in Table 1 may be involved in the epigenetic regulation of genes involved in the cell cycle (AURKB, CDC45, and CDK6), cell proliferation (EGFR and VEGFA), and angiogenesis (VEGFA), contributing to tumor growth. Huang et al. experimentally proved that upregulation of AURKB in H1975 cells showed a positive correlation with the expression of hsa-miR-93-5p, hsa-miR-17-5p, and hsa-miR-130b-3p [34]. Liu et al. demonstrated CDC45 regulation by miR-485-5p [35].

Other genes (AJAP1, MMP9, and STC2) promote metastasis formation by controlling cell adhesion or migration. Adherens junction-associated protein 1 (AJAP1), a putative tumor suppressor downregulated in GPs based on mRNA NGS, is an experimentally validated target of hsa-miR-196a-5p [26]. Stanniocalcin-2 (STC2) is a secreted glycoprotein that has been shown to play a role in the development of cancer through its upregulation. Yun et al. experimentally proved that STC2 elevates the expression level of matrix metalloproteinases (MMPs) via the Mitogen-Activated Protein Kinase (MAPK) signaling pathway [73]. STC2 expression is regulated by hsa-miR-381-3p [29], while hsa-miR-490-3p led to a notable reduction in MMP9 expression [27].

In addition, the regulation of tumor-associated macrophages (LTBP-1 and POSTN) could make the tumor microenvironment more favorable for the spread of cancer cells. Periostin (POSTN) is a secreted extracellular matrix protein that plays a role in attracting M2-type tumor-associated macrophages (TAMs), which are monocyte-derived macrophages from peripheral blood, to facilitate the growth of GBM [74]. Liu et al. showed that miR-340-5p plays an important role in the regulation of TAMs by targeting POSTN and LTBP-1 [28].

The results of miRNA and mRNA NGS were used to demonstrate the relationship between the two datasets and demonstrate their involvement at different stages of the tumorigenesis process in GBM.

## 4. Materials and Methods

### 4.1. Patients and Samples

GPs were identified and handled at the Department of Neurosurgery, Faculty of Medicine, University of Debrecen, Hungary. Written informed consent was obtained from each member of the trial groups. The demographic and clinical data of GPs were collected from the medical record overview. The patients—all of them IDH wild type—did not receive chemotherapy or radiation therapy before participating in this study. The ages of GPs were between 57 and 70 years, with a mean age of 63 years, while controls were aged between 52 and 80 years, with a mean age of 64 years, selected for miRNA and RNA NGS (Table 2). In the case of 28 members of the control group, the age ranged between 38 and 78, with a median age of 63, while in the case of 30 GPs involved in the validation group, the age ranged between 37 and 80, with a median age of 61. The number of women and men involved in the validation procedure was equal in the control and patient groups as well. The study was approved by the Scientific and Research Ethics Committee of the Medical Research Council of the Ministry of Health, Budapest, Hungary (ETT TUKEB; project identification code: IV/1753-/2021/EKU) and was conducted in accordance with the Declaration of Helsinki, and each patient signed the consent form. Tissue samples were collected from the peripheral tumor region of 5 individuals diagnosed with lower-grade (grade 1–2) glioma but whose histology proved peripheral tissue without tumor cells serving as a control group, and from 5 patients with GBM. All samples of the participating individuals were confirmed histopathologically. These intraoperative quick-frozen tissue samples were stored at −80 °C until further processing.

### 4.2. Tissue Exploration and RNA Isolation and Purification for Next-Generation Sequencing (NGS)

Tissue samples were dissected on ice, then disrupted and homogenized by the MagNa Lyser instrument (Roche Ltd., Basel, Switzerland) for purification of total RNA (including small RNAs) using the miRNeasy Mini Kit (Qiagen, Hilden, Germany). Each step was performed according to the manufacturer’s instructions. The amount of the tissue sample was 30 mg in each case. The quality of isolated RNA was analyzed by a Nanodrop spectrophotometer (Thermo Scientific, Waltham, MA, USA).

### 4.3. Next-Generation Sequencing (NGS) and Determination of Differentially Expressed (DE) miRNAs and mRNAs

To obtain global miRNA and mRNA transcriptome data, high-throughput miRNA and mRNA sequencing analysis was performed on the Illumina NextSeq 500 sequencing platform. The sequencing process, from library preparation to raw data analysis, was performed by the Genomic Medicine and Bioinformatics Core Facility (Department of Biochemistry and Molecular Biology, Faculty of Medicine, University of Debrecen). Six replicates were used for sequencing from both the control and patient groups. The quality of RNA samples was determined by the Agilent Bioanalyzer with the Eukaryotic Total RNA Nano Kit, following the instructions of the manufacturer. For library preparation, those samples were selected in which the value of the RNA integrity number (RIN) was higher than seven. Small RNA-Seq libraries were prepared from 1 µg total RNA using the NEBNext Multiplex Small RNA Prep Set for Illumina (1-48) 96 rxn kit (New England BioLabs, Ipswich, MA, USA), according to the manufacturer’s protocol. RNA-Seq libraries were generated from rRNA-depleted samples (NEBNext^®^ rRNA Depletion Kit, New England BioLabs) using the Ultra II RNA Sample Prep Kit (New England BioLabs) according to the manufacturer’s protocol. The fragment size distribution and molarity of libraries were checked on the Agilent Bioanalyzer DNA1000 chip (Agilent Technologies, Santa Clara, CA, USA). Then, a single read 50 bp sequencing run (in case of miRNA sequencing) and single-end 75 cycle sequencing (in case of mRNA sequencing) were performed using the Illumina NextSeq 500 instrument (Illumina, San Diego, CA, USA). Raw miRNA sequencing data (fastq) were aligned to the human reference genome version GRCh38 using the Novoalign algorithm, while raw mRNA sequencing data (fastq) were aligned to the human reference genome version GRCh38 using the HISAT2 algorithm, and BAM files were generated. Downstream analysis was performed using StrandNGS software (version 2.8, build 230243; Strand Life Sciences, Bangalore, India) (www.strand-ngs.com), which involved normalization by applying the BAM files using the DESeq algorithm.

The normalized values of DE miRNAs and mRNAs were used to calculate the extent of changes in miRNA and mRNA expression levels between GPs and the control group. The evaluation of miRNA and mRNA expression data was performed using the iDEP.96 tool (http://bioinformatics.sdstate.edu/idep96; accessed on 28 November 2022). In the preprocessing step, miRNAs or mRNAs with low or missing expression values were filtered out (≥CPM 1 in all libraries). Bioinformatics analysis included hierarchical clustering with a heatmap, unconditional k-means clustering, PCA, and MA plots, applying the iDEP.96 web tool as well. For differential expression analysis, the DESeq2 package of iDEP was used. Significantly deregulated miRNAs and mRNAs were identified after adjusting the cut-off values for a fold change (FC) ≥ 2 and a false discovery rate (FDR) ≤ 0.05.

### 4.4. Prediction of Targets of Differentially Expressed (DE) miRNAs, Construction of PPI Networks, Gene Ontology (GO), and Functional Annotation and Pathway Enrichment Analysis

Experimentally validated target genes of DE miRNAs were identified by the web-based miRNet (http://www.mirnet.ca; accessed on 12 December 2022) and miRNA Enrichment Analysis and Annotation (miEAA) tools, both using the miRTargetLink database (https://ccb-compute.cs.uni-saarland.de/mirtargetlink2/) for target prediction. The miRNA–target gene network was constructed with the miRNet tool, and the top miRNAs were identified by their degree-centrality values.

Protein–protein interaction (PPI) networks were also constructed using the 50 most upregulated and downregulated mRNAs, applying the NetworkAnalyst 3.0 tool (www.NetworkAnalyst.ca), and hub proteins were ranked by their degree-centrality values.

The network-based GO and functional enrichment and pathway analysis were carried out by the NetworkAnalyst 3.0 tool using the KEGG database options of the tool for pathway enrichment analysis. A *p*-value of <0.05 was considered statistically significant in the analysis.

### 4.5. Validation of miRNA-Seq Results by Quantitative Real-Time PCR (RT-qPCR)

For the validation process, total RNA was extracted from 30 mg tissue samples of 28 control persons and 30 GBM patients using the miRNeasy Mini Kit (Qiagen, Hilden, Germany) according to the manufacturer’s manual. The concentration and quality of purified RNA in each sample were measured by a Nanodrop spectrophotometer (Thermo Scientific, Waltham, MA, USA). To detect and measure the number of mature miRNAs, the miRCURY LNA miRNA PCR Assay (Qiagen, Hilden, Germany) was applied. For the reverse transcription of miRNAs, the miRCURY LNA RT Kit (Qiagen, Hilden, Germany) was used. The reverse transcription reaction happened at 42 °C for 60 min, followed by inactivation of the reverse transcriptase enzyme at 95 °C for 5 min. The quantitative real-time PCR reaction was completed using the LightCycler^®^ 96 instrument (Roche Ltd., Pleasanton, CA, USA) and the miRCURY LNA SYBR Green PCR Kit (Qiagen, Hilden, Germany) in order to identify the expression level of hsa-miR-196a-5p, hsa-miR-21-3p, hsa-miR-10b-3p, hsa-miR-383-5p, and hsa-miR-490-3p in both GPs and control samples. The circumstances for the PCR reactions were the following: initial heat activation at 95 °C for 120 s, followed by 45 two-step amplification cycles (denaturation at 95 °C for 10 s and combined annealing/extension at 56 °C for 60 s). Ultimately, melting curves were generated by fluorescent measurements at 95 °C for 20 s, 40 °C for 20 s, and 85 °C for 1 s. The PCR reaction was terminated by a cooling step at 37 °C for 30 s. The expression levels of the investigated miRNAs were calculated using the comparative cycle threshold (Ct) method, and hsa-miR-103a-3p was chosen as the internal control. The fold change was measured by the equation ∆Ct, and ∆Ct was calculated by subtracting the mean Ct value of hsa-miR-103a-3p from the Ct value of the target miRNA.

### 4.6. Validation of mRNA-Seq Results by Quantitative Real-Time PCR (RT-qPCR)

To validate the mRNA sequencing results, the same total RNA samples were employed as in the case of validating the miRNA sequencing data, as previously described. A total of 500 ng of RNA was used as a template during the reverse transcription process, which was carried out using the Maxima First Strand cDNA Synthesis Kit (Thermo Fisher Scientific, Waltham, MA, USA) in accordance with the instructions provided by the manufacturer. A concentration of total RNA and cDNA was measured by a NanoDrop LITE Spectrophotometer (Thermo Fisher Scientific, Waltham, MA, USA). The expression of E2F2, HOXD13, VEGFA, CDC45, AURKB, HOXC10, MYBL2, FABP6, PRLHR, NEUROD6, CBLN1, HRH3, HCN1, and RELN was determined by qPCR using the Maxima™ SYBR Green qPCR Master Mix (Thermo Fisher Scientific, Waltham, MA, USA), applying a Lightcycler 96 instrument (Roche, Pleasanton, CA, USA), following the instructions of the manufacturer. The sequences of forward and reverse primers are included in Supplementary Materials Table S3. As an endogenous control gene, GAPDH was used for normalization of the mRNA expression values. Each RT-qPCR reaction was performed in triplicate. Fold changes (FCs) are presented in a log2 scale.

### 4.7. Statistical Analysis

In the case of the RT-qPCR validation of miRNAs and mRNAs, the distribution of data was analyzed using the Kolmogorov–Smirnov test. The statistical significance of expression values was calculated by the nonparametric Mann–Whitney U test using the GraphPad Prism 8.0.1 program. The difference in miRNA and mRNA expression was considered significant at *p* < 0.05. ROC-AUC graphs were created with easyROC curve analysis (ver. 1.3.1.) (http://biosoft.erciyes.edu.tr/app/easyROC/), accessed on 5 February 2021. ROC analysis was performed to determine the optimal cut-off points based on the best balance of sensitivity and specificity. The significance level was *p* < 0.05 in the case of all tests.

## 5. Conclusions

The detection and quantification of miRNAs and mRNAs in tissue samples of cancer patients can be of diagnostic and prognostic significance. In our study applying miRNA and mRNA NGS followed by RT-qPCR in validation processes, a miRNA panel composed of hsa-miR-196a-5p, hsa-miR-21-3p, hsa-miR-10b-3p, hsa-miR-383-5p, and hsa-miR-490-3p and an mRNA panel, including E2F2, HOXD13, VEGFA, CDC45, AURKB, HOXC10, MYBL2, FABP6, PRLHR, NEUROD6, CBLN1, HRH3, HCN1, and RELN, was constructed that could be helpful in the tissue sample-based diagnosis of GBM in the Hungarian population. Our functional annotation analysis shows that experimentally validated targets of DE miRNAs are key regulators of tumor formation, suggesting that miRNAs might play an important pathophysiological role in the formation of different tumor types. A clear limitation of our study is the low sample size. Although our results are statistically significant, further studies are needed in independent cohorts to confirm their utility and potential clinical application. GBM was one of the first tumor types to be analyzed by The Cancer Genome Atlas (TCGA) consortium. We took advantage of the Glioblastoma Multiforme (TCGA-GBM) data collection to further validate the credibility and novelty of our miRNA set and investigated the overlap between our miRNAs and GBM-associated miRNAs present in the TCGA database (and TCGA-based publications). We found that a reduced set of miRNAs (45 out of the 117, Table S1) analyzed here were represented in these resources. Not surprisingly, the key genes identified as frequently mutated genes in GBM—e.g., EGFR, TP53, MYC, and TERT—were present among the target genes of our set of deregulated miRNAs, highlighting the regulatory role of miRNAs in the progression of this disease. The network-based pathway enrichment analysis of our set of dysregulated miRNAs identifies core pathways, like the FoxO signaling pathway, that are considered obligatory events in most GBM tumors. Our results suggest that miRNA and mRNA signatures might help to better understand tumor development; furthermore, they could be diagnostic indicators of GBM and, thus, potentially predict the clinical status of individuals.

## Figures and Tables

**Table 1:** Correlation between the deregulated miRNAs and their experimentally validated target genes found in our NGS dataset.

Regulated Gene	Biological Process	Expression Status (Up/Down)	miRNA	Expression Status (Up/Down)	References
AHR	regulation of transcription	up	hsa-mir-124-3p	down	[19]
E2F2	regulation of transcription	up	hsa-mir-218-5p	down	[20]
HOXC10	regulation of transcription	up	hsa-mir-129-5p	down	[21]
HOXD4	regulation of transcription	up	hsa-mir-10b	up	[22,23]
MYBL2	regulation of transcription	up	hsa-mir-30e-5p	down	[24]
NEUROD2	regulation of transcription	down	hsa-mir-210-3p	up	[25]
AJAP1	cell adhesion	down	hsa-mir-196a-5p	up	[26]
MMP9	cell migration	up	hsa-mir-490-3p	down	[27]
POSTN	cell migration	up	hsa-mir-340-5p	down	[28]
STC2	cell migration	up	hsa-mir-381-3p	down	[29]
GABRA1	synaptic transmission	down	hsa-mir-155 -5p	up	[30]
GABRB2	synaptic transmission	down	hsa-mir-10a-5p hsa-mir-10b-5p	up	[31,32]
HCN1	transmission of nerve impulse	down	hsa-mir-10a-5p hsa-mir-10b-5p	up	[33]
AURKA	cell cycle regulation	up	hsa-mir-124-3p	down	[34]
AURKB	cell cycle regulation	up	hsa-miR-93-5p, hsa-miR-17-5p, hsa-miR-130b-3p	up	[34]
CDC45	cell cycle regulation	up	hsa-mir-485-5p	down	[35]
CDK6	cell cycle regulation	up	hsa-mir-107	down	[36]
EGFR	cell proliferation	up	hsa-mir-7	down	[37]
VEGFA	cell proliferation angiogenesis	up	hsa-mir-383-5p	down	[38]
LTBP-1	regulation of tumor-associated macrophages	up	hsa-mir-340-5p	down	[28]
POSTN	regulation of tumor-associated macrophages	up	hsa-mir-340-5p	down	[28]
BCL2	apoptosis	up	hsa-mir-136-3p	down	[39]
EZH2	epigenetic regulation	up	hsa-miR-138-5p, hsa-miR-490-3p	down	[40,41]

**Table 2:** Information about the gender, age, and immunohistochemical characteristics of 5 control and 5 GBM patients who took part in NGS (a) and the 28 control and 30 GBM patients involved in the RT-qPCR validation group (b).

(a)			
**Members of NGS**	**Gender**	**Age**	**Immunohistochemical characteristics**
Control_1	M	70	-
Control_2	M	52	-
Control_3	M	52	-
Control_4	F	71	-
Control_5	F	80	-
GBM_1	M	65	IDH wild type
GBM_2	M	57	IDH wild type
GBM_3	M	70	IDH wild type-
GBM_4	F	60	IDH wild type-
GBM_5	F	56	IDH wild type-
(b)			
**Characteristic**	**Control Group (number)**		**GBM Group (number)**
Sex (M/F)	14/14		15/15
Age			
Median	63		61
Range	38–78		37–80

## Data Availability

The data presented in this study are openly available in the Gene Expression Omnibus (GEO) database (https://www.ncbi.nlm.nih.gov/geo/, accessed on 21 December 2024, at the accession numbers GSE244332 and GSE285290.

## Supplementary Materials

The following supporting information can be downloaded at: https://www.mdpi.com/article/10.3390/ph18030431/s1, Table S1: The whole list of the 117 deregulated miRNAs; Table S2: The whole list of deregulated mRNAs; Table S3: The list of primer sequences used for validation of differentially expressed mRNAs by RT-qPCR.
