# Supplementary material for: Identification of Deregulated miRNAs and mRNAs Involved in Tumorigenesis and Detection of Glioblastoma Patients Applying Next-Generation RNA Sequencing
Source: Pharmaceuticals (Basel). 2025 Mar 19;18(3):431. doi: 10.3390/ph18030431 (PMC11944724; doi:10.3390/ph18030431)
Supplement: Supplementary file 1 [file pharmaceuticals-18-00431-s001.zip › Table S1. The whole list of the 117 deregulated miRNAs.pdf]

Regulation	miRNA	logFC
Up	HSA-MIR-10B-5P	6.92074574666667
Up	HSA-MIR-196A-5P	5.62050166666667
Up	HSA-MIR-10A-5P	5.48007084333334
Up	HSA-MIR-21-3P	4.39117474
Up	HSA-MIR-92B-5P	3.90039191133333
Up	HSA-MIR-21-5P	3.71833013333334
Up	HSA-MIR-210-3P	3.67009291666667
Up	HSA-MIR-10B-3P	3.66695223333333
Up	HSA-MIR-503-5P	3.47447538866667
Up	HSA-MIR-450A-5P	3.387407228
Up	HSA-MIR-217-5P	3.34686528133333
Up	HSA-MIR-155-5P	3.30287179666667
Up	HSA-MIR-424-5P	3.24971973333333
Up	HSA-MIR-542-3P	3.07204993333334
Up	HSA-MIR-424-3P	2.99402544533334
Up	HSA-MIR-27A-3P	2.66789120666667
Up	HSA-MIR-23A-3P	2.55821536333333
Up	HSA-MIR-16-1-3P	2.51823068333333
Up	HSA-MIR-130A-3P	2.22494305333333
Up	HSA-MIR-15B-3P	2.07992479666667
Up	HSA-MIR-339-5P	2.0315354
Up	HSA-MIR-15A-5P	2.0115549
Up	HSA-MIR-92B-3P	1.98302636666667
Up	HSA-MIR-106B-5P	1.94801386666667
Up	HSA-MIR-130B-3P	1.9224647
Up	HSA-MIR-19B-3P	1.86265629333334
Up	HSA-MIR-34A-5P	1.84692903666667
Up	HSA-MIR-200A-3P	1.71856434666667
Up	HSA-LET-7I-3P	1.68810876
Up	HSA-MIR-4677-3P	1.66613401666667
Up	HSA-MIR-429	1.57553668666667
Up	HSA-MIR-28-3P	1.42726913333333
Up	HSA-MIR-25-3P	1.40157130000001
Up	HSA-MIR-93-5P	1.32426601666667
Up	HSA-MIR-17-5P	1.27476113333334

Regulation	miRNA	logFC
Down	HSA-MIR-383-5P	-6.33919243333333
Down	HSA-MIR-129-5P	-5.96239611666666
Down	HSA-MIR-129-2-3P	-5.85958781666666
Down	HSA-MIR-219A-2-3P	-5.80101541666667
Down	HSA-MIR-218-5P	-5.75067083333333
Down	HSA-MIR-490-3P	-5.61557593
Down	HSA-MIR-504-5P	-5.21483623
Down	HSA-MIR-124-3P	-4.89750279666667
Down	HSA-MIR-129-1-3P	-4.75589369
Down	HSA-MIR-885-5P	-4.63809322666666
Down	HSA-MIR-330-5P	-4.60626233
Down	HSA-MIR-433-3P	-4.52174226666666
Down	HSA-MIR-139-5P	-4.50872421666667
Down	HSA-MIR-138-5P	-4.34196468
Down	HSA-MIR-139-3P	-4.30376213333333
Down	HSA-MIR-128-3P	-3.99113566666666
Down	HSA-MIR-124-5P	-3.90731508666667
Down	HSA-MIR-184	-3.89179219
Down	HSA-MIR-874-3P	-3.87094955666667
Down	HSA-MIR-487B-3P	-3.77544195
Down	HSA-MIR-330-3P	-3.63640526
Down	HSA-MIR-1249-3P	-3.51889316
Down	HSA-MIR-1224-3P	-3.46251966
Down	HSA-MIR-539-3P	-3.45594785666666
Down	HSA-MIR-1197	-3.45575897
Down	HSA-MIR-1250-5P	-3.41333333333333
Down	HSA-MIR-539-5P	-3.37122667333333
Down	HSA-MIR-656-3P	-3.35843951333333
Down	HSA-MIR-329-3P	-3.32080248333333
Down	HSA-MIR-346	-3.25997975333333
Down	HSA-MIR-7-5P	-3.23231116666666
Down	HSA-MIR-1296-5P	-3.12185100666666
Down	HSA-MIR-514A-3P	-3.11427419
Down	HSA-MIR-873-3P	-2.95810569
Down	HSA-MIR-431-3P	-2.95187304333333
Down	HSA-MIR-889-3P	-2.9249674
Down	HSA-MIR-338-3P	-2.8647302
Down	HSA-MIR-219A-5P	-2.85449067
Down	HSA-MIR-487A-5P	-2.79621862333333
Down	HSA-MIR-584-5P	-2.76451488
Down	HSA-MIR-491-5P	-2.74336322
Down	HSA-MIR-7-1-3P	-2.71952808
Down	HSA-MIR-1298-5P	-2.70512398
Down	HSA-MIR-377-5P	-2.68226115

Down	HSA-MIR-3200-3P	-2.64483777333333
Down	HSA-MIR-628-5P	-2.63634885
Down	HSA-MIR-495-3P	-2.5976953
Down	HSA-MIR-485-5P	-2.57975563
Down	HSA-MIR-381-3P	-2.564575
Down	HSA-MIR-873-5P	-2.53085096
Down	HSA-MIR-410-3P	-2.47960759
Down	HSA-MIR-411-5P	-2.4551021
Down	HSA-MIR-95-3P	-2.42314064
Down	HSA-MIR-105-5P	-2.37356112
Down	HSA-MIR-29C-5P	-2.30568902
Down	HSA-MIR-885-3P	-2.24787286
Down	HSA-MIR-1255A	-2.24741969333333
Down	HSA-MIR-499A-5P	-2.2221474
Down	HSA-MIR-340-3P	-2.18616651
Down	HSA-MIR-379-5P	-2.15981985
Down	HSA-MIR-107	-2.15260868333333
Down	HSA-MIR-136-3P	-2.11886626666666
Down	HSA-MIR-326	-2.10146139333333
Down	HSA-MIR-138-2-3P	-2.098848948
Down	HSA-MIR-598-3P	-2.05096456666666
Down	HSA-MIR-3943	-1.9934682
Down	HSA-MIR-541-3P	-1.98500026
Down	HSA-MIR-433-5P	-1.76596662533333
Down	HSA-MIR-1298-3P	-1.7089049
Down	HSA-MIR-509-3-5P	-1.67768108
Down	HSA-MIR-488-5P	-1.67070325666667
Down	HSA-MIR-328-3P	-1.66700259999999
Down	HSA-MIR-31-3P	-1.63139764666667
Down	HSA-MIR-369-5P	-1.607546086
Down	HSA-MIR-3912-3P	-1.5639192
Down	HSA-MIR-4787-3P	-1.42311268333333
Down	HSA-MIR-769-5P	-1.39808531666666
Down	HSA-MIR-340-5P	-1.30384033333333
Down	HSA-MIR-6866-5P	-1.25151838866667
Down	HSA-MIR-5584-5P	-1.118054286
Down	HSA-MIR-744-5P	-1.11605161
Down	HSA-MIR-338-5P	-1.029708072
