# Supplementary material for: Identification of Deregulated miRNAs and mRNAs Involved in Tumorigenesis and Detection of Glioblastoma Patients Applying Next-Generation RNA Sequencing
Source: Pharmaceuticals (Basel). 2025 Mar 19;18(3):431. doi: 10.3390/ph18030431 (PMC11944724; doi:10.3390/ph18030431)
Supplement: Supplementary file 1 [file pharmaceuticals-18-00431-s001.zip › Table S2. The whole list of deregulated mRNAs (1).pdf]

Regulation	mRNA	logFC
Up	HOXD10	7.0657492
Up	SHOX2	6.8692629125
Up	POSTN	6.09422871
Up	TOP2A	6.0893005275
Up	HOXD11	5.95118
Up	HOXB3	5.941146115
Up	EN1	5.9337394
Up	HOXD9	5.82288678
Up	MKI67	5.741333395
Up	MYBL2	5.7330838375
Up	MEOX2	5.58859493
Up	STC2	5.380600925
Up	MMP9	5.3675641975
Up	NNMT	5.361172555
Up	ESPL1	5.2765701725
Up	ABCC3	5.18728671
Up	RRM2	5.0230804625
Up	TROAP	5.007641365
Up	CNGA3	4.991051825
Up	HOXC10	4.97072522
Up	FCGBP	4.93623435
Up	NKX2-5	4.9147794175
Up	GPX8	4.87912896
Up	KIF18B	4.848567365
Up	PLEKHA4	4.810283925
Up	BUB1	4.8051585975
Up	HOXD3	4.800507535
Up	APOL4	4.7762468325
Up	IGFBP2	4.770905175
Up	DLGAP5	4.76318599
Up	CENPF	4.7547078
Up	SIX1	4.73823713
Up	UHRF1	4.722056395
Up	CDCA2	4.6846211625
Up	CPXM1	4.6341451875
Up	AURKB	4.60474168
Up	IGF2BP3	4.57846774
Up	KNL1	4.54865113
Up	METTL7B	4.543538125
Up	COL4A1	4.522256625
Up	GTSE1	4.5156906375
Up	FOXJ1	4.511366245
Up	LIF	4.49293964
Up	TNC	4.4566955

Up	TGFBI	4.37856445
Up	UBE2C	4.3717389375
Up	NDC80	4.3698178775
Up	LHX9	4.3471907175
Up	KIF14	4.3430560075
Up	EGFR	4.331659675
Up	CA3	4.321852155
Up	CLEC5A	4.3146537875
Up	CKAP2L	4.3134822775
Up	CDC45	4.31232673
Up	VEGFA	4.308474775
Up	HAS2	4.3021974
Up	HOXC4	4.2792974575
Up	ASPM	4.275999295
Up	PRPH	4.273429215
Up	PTX3	4.2617011375
Up	HJURP	4.25461533
Up	MELK	4.233870305
Up	SEC61G	4.23323736
Up	PITX1	4.1629158975
Up	F2R	4.15841434
Up	SOX11	4.12669595
Up	TBX15	4.108832665
Up	NES	4.10766095
Up	VIM	4.080144725
Up	COL4A2	4.0720936
Up	IQGAP3	4.069798355
Up	HOXD8	4.06295478
Up	DPEP1	4.05861738
Up	RGS1	4.052365625
Up	LAMB4	4.0387621625
Up	CELSR1	4.029367395
Up	ADAMTS15	4.00583126
Up	NMB	4.004004245
Up	FN1	3.99036605
Up	NUSAP1	3.98086828
Up	KIFC1	3.9805313575
Up	MRC2	3.94620235
Up	LOX	3.930950765
Up	HLA-B	3.9165129
Up	DMRTA2	3.91578177
Up	C6orf118	3.907707765
Up	GSC	3.888107096
Up	SRPX2	3.872495115
Up	AGMO	3.862640035

Up	BUB1B	3.85593241
Up	CDCA7	3.854891355
Up	E2F7	3.8474414775
Up	ADM	3.843907515
Up	MCM10	3.83808836
Up	TMEM71	3.824802485
Up	AEBP1	3.817266475
Up	NCAPH	3.810941345
Up	ID3	3.810932175
Up	CEP55	3.80122487
Up	TXLNB	3.78633323
Up	CENPK	3.7568793425
Up	MMP14	3.747550475
Up	C1R	3.7415319
Up	KIF18A	3.736945425
Up	HOXD4	3.72406136
Up	ANXA1	3.712157875
Up	EVC2	3.71095253
Up	HOXD13	3.69889674
Up	FMOD	3.69278089
Up	MUC1	3.69199859
Up	ANXA2	3.68915865
Up	BATF2	3.6730301725
Up	TPX2	3.65574295
Up	TIMP1	3.6148791
Up	HLA-A	3.60677195
Up	E2F8	3.6058479475
Up	E2F2	3.59822102
Up	KIF4A	3.58448582
Up	TMEM45A	3.5669979325
Up	TNFRSF19	3.55851642
Up	KIAA0040	3.551492925
Up	OTP	3.5465570775
Up	GBP1	3.536066825
Up	CD44	3.534805625
Up	EMILIN1	3.52760865
Up	PLP2	3.481898475
Up	S100A10	3.47476795
Up	GDF15	3.471687505
Up	WEE1	3.464191995
Up	KLHDC8A	3.46282201
Up	DRAXIN	3.455048295
Up	BIRC5	3.443115435
Up	IGFBP5	3.427964575
Up	TTK	3.403961155

Up	SKA3	3.39905818
Up	ESM1	3.3582230575
Up	TNFAIP6	3.3424736875
Up	PLAU	3.3315551
Up	HOXC9	3.31581092
Up	TEAD2	3.30625698
Up	PDPN	3.30301845
Up	MDFI	3.300851785
Up	HS3ST3A1	3.299613795
Up	PXDN	3.293128625
Up	EMP3	3.258102255
Up	GAS1	3.253943745
Up	TGFB1I1	3.235992485
Up	TERT	3.23485864
Up	MACC1	3.23327676
Up	AJUBA	3.227771855
Up	F2RL1	3.2267239225
Up	APOC1	3.22662565
Up	KCNE4	3.226415685
Up	S1PR3	3.21738515
Up	SPC24	3.21073366
Up	CDKN2C	3.2062923
Up	HSPG2	3.20089775
Up	HILPDA	3.1992479
Up	EXO1	3.19430905
Up	KIF2C	3.192255375
Up	HOXA7	3.1818856
Up	SERPINH1	3.175263375
Up	SPATA12	3.173598945
Up	CA12	3.173235905
Up	IFI30	3.170732785
Up	CD101	3.168689675
Up	PLEKHG2	3.1659301
Up	ADAM12	3.157273725
Up	EDA2R	3.15625385
Up	BTN3A2	3.14893414
Up	DIAPH3	3.140926135
Up	C1RL	3.127257785
Up	OSR2	3.12592376
Up	TWIST1	3.109073755
Up	GABRR2	3.1024995675
Up	MCUB	3.09697865
Up	STAB1	3.09672675
Up	RAB20	3.092145475
Up	TAGLN2	3.08801915

Up	FKBP10	3.086555825
Up	FLNA	3.085601
Up	CDK1	3.07921718
Up	EXOC3L2	3.067968305
Up	CCDC80	3.0665228
Up	PYGL	3.055824805
Up	PRRX1	3.054233225
Up	ORC1	3.03514669
Up	TNFRSF12A	3.0309278
Up	ASF1B	3.0090595075
Up	S100A3	3.0036164725
Up	BGN	2.997843925
Up	NID1	2.997022165
Up	C21orf62	2.99416022
Up	RDH10	2.981971925
Up	HOXB6	2.96938626
Up	PGF	2.968779455
Up	RUNX1	2.961291025
Up	SLC5A9	2.9379386
Up	GLIS3	2.929798975
Up	LFNG	2.9235932
Up	CLEC18B	2.921453895
Up	BRCA2	2.921310305
Up	SAMD9L	2.91788115
Up	ESCO2	2.91458069
Up	ADAMTS7	2.91117239
Up	STEAP3	2.904755795
Up	VWA3B	2.89157614
Up	CRISPLD1	2.88287234
Up	TEAD4	2.882671715
Up	BCL3	2.87374501
Up	ADAMTS6	2.86771045
Up	SLC11A1	2.85642645
Up	CHRNA9	2.853806255
Up	SYTL4	2.853623225
Up	ADAM33	2.853186285
Up	SYDE1	2.8473885
Up	MEX3A	2.84395416
Up	CDC25A	2.8421116375
Up	CD93	2.8384397
Up	CEBPD	2.835310325
Up	CCDC169	2.831395365
Up	IL23A	2.8305394
Up	TFPI	2.82743341
Up	TACC3	2.82023263

Up	SLC28A1	2.81656167
Up	SNAI2	2.81386999
Up	TP53	2.81338546
Up	IKBIP	2.80818337
Up	SLFN12	2.80817763
Up	TMEM176A	2.80388426
Up	PLEK2	2.7961872575
Up	RCN3	2.795471705
Up	KIF15	2.79418975
Up	NLRC5	2.78302492
Up	IGDCC4	2.77916049
Up	SPARC	2.763869325
Up	TBX2	2.75864468
Up	RAD51	2.7575512925
Up	WWTR1	2.7560105
Up	APLN	2.753004425
Up	SPC25	2.7505407775
Up	CKS2	2.72837613
Up	EZH2	2.72794667
Up	ZBTB42	2.72430249
Up	TRIM22	2.718229475
Up	SYTL3	2.71150579
Up	SLC43A3	2.70364913
Up	ST14	2.700305475
Up	SIGLEC7	2.69300565
Up	IGSF9	2.6917344775
Up	CDCA7L	2.686493835
Up	ANXA5	2.683382875
Up	TRIP10	2.677906415
Up	HK2	2.675981725
Up	CXCR4	2.670968445
Up	LVRN	2.6704865075
Up	MDK	2.660454235
Up	S100A4	2.6593319
Up	SMO	2.647963675
Up	SOX4	2.647761875
Up	PARP9	2.631599875
Up	FBP1	2.6263961675
Up	EPHB4	2.623637695
Up	FSTL1	2.62262165
Up	TICRR	2.61762843
Up	SMIM3	2.616645555
Up	PTGIR	2.616152455
Up	ACKR3	2.615661235
Up	CENPU	2.61532131

Up	GPR84	2.6146931275
Up	CDK2	2.594054085
Up	IQGAP2	2.588366475
Up	DTX3L	2.58436455
Up	PCDHB9	2.5827848
Up	ITGA5	2.58098461
Up	TRIM5	2.58058509
Up	TMEM176B	2.57890189
Up	TMEM255A	2.577672935
Up	FAM20C	2.577084625
Up	LRRC25	2.576327525
Up	PABPC1L	2.569301425
Up	LDLRAD2	2.56482064
Up	CSRP2	2.563160665
Up	PTTG1	2.555464415
Up	PLOD2	2.555448525
Up	PCDHB16	2.55321187
Up	PLTP	2.54999225
Up	PPP1R13L	2.549865915
Up	HELZ2	2.54869805
Up	SERPING1	2.542340775
Up	ZNF217	2.540693725
Up	NKX3-2	2.53673126
Up	LHCGR	2.53289166
Up	S100A6	2.528992305
Up	CBX2	2.52896945
Up	PCDHB7	2.528050765
Up	CD99	2.5263296
Up	JAG1	2.52538175
Up	NTN1	2.520798825
Up	SAMD9	2.51704902
Up	PLSCR1	2.514506355
Up	RHOC	2.513181575
Up	SPSB4	2.511555025
Up	ONECUT1	2.5091076075
Up	CASP1	2.500100115
Up	CDK6	2.494618525
Up	FOXD1	2.494449365
Up	HPD	2.48418066
Up	PALLD	2.4792684
Up	BMF	2.47878302
Up	MFNG	2.47445767
Up	SPATA13	2.4736554025
Up	PCDHB1	2.470410675
Up	APOL6	2.468719625

Up	IFI16	2.46815835
Up	MGAM	2.468039565
Up	NEIL3	2.465718175
Up	POU3F4	2.464371385
Up	HS3ST3B1	2.458012135
Up	SLC2A10	2.4552785
Up	ZFP36L2	2.449145875
Up	SP140L	2.447137845
Up	TGIF1	2.44421344
Up	CCDC102A	2.442341505
Up	FXYD2	2.44034118
Up	VCAN	2.435543
Up	OSMR	2.434111775
Up	PLK4	2.43288947
Up	NOTCH3	2.432012425
Up	ADAMTS9	2.422341975
Up	FANCD2	2.421535845
Up	TEKT2	2.410041375
Up	GDPD2	2.40536214
Up	TXNDC2	2.395806185
Up	FNDC3B	2.3954034
Up	CLEC18A	2.38850165
Up	SP100	2.38842715
Up	CCDC103	2.38812122
Up	CD248	2.380473235
Up	TRIP6	2.37688955
Up	KIF7	2.37300718
Up	CXCL11	2.36866146
Up	C1orf226	2.36582437
Up	ITGB3	2.356784735
Up	PGGHG	2.354425475
Up	NMI	2.3527884
Up	PCDHGA1	2.35057632
Up	TRIM6	2.3486756225
Up	ABI3	2.34305783
Up	FAM187A	2.34182631
Up	OAS3	2.3413296
Up	DNAAF3	2.324110535
Up	PRR15	2.3236844375
Up	PPP1R3B	2.32097653
Up	EIF4EBP1	2.31848644
Up	CASP4	2.315827235
Up	KIF11	2.31102906
Up	NCF4	2.308586515
Up	SERINC2	2.30602703

Up	AOC1	2.3052884525
Up	TGIF2	2.29954656
Up	GLIPR2	2.298991225
Up	CSPG4	2.29586959
Up	PRDX4	2.29539407
Up	GSX2	2.29501402
Up	VCAM1	2.292554265
Up	APOBEC3G	2.290949615
Up	ACRV1	2.28851704
Up	RAD51AP1	2.288028135
Up	TGM5	2.2877097
Up	PLIN2	2.287098905
Up	STON1-GTF2A1L	2.28425997
Up	ARHGAP11A	2.2842459
Up	ABCA1	2.2836892
Up	FHL3	2.28277992
Up	YBX3	2.28267809
Up	TCAF2	2.28034952
Up	TNFRSF10B	2.27896614
Up	LAMB2	2.27527205
Up	TRIM56	2.273071
Up	TCIRG1	2.271984885
Up	EFEMP1	2.26768371
Up	GSDMD	2.26636367
Up	LPL	2.261950325
Up	C2orf66	2.2612181475
Up	AFAP1L1	2.25723298
Up	DNALI1	2.25593628
Up	CLEC18C	2.255749045
Up	PCDHB3	2.25052181
Up	TM6SF2	2.2492891925
Up	OAS1	2.24831843
Up	GINS4	2.24509598
Up	BTN3A1	2.242791375
Up	MASP1	2.241415785
Up	DNAJC22	2.23976076
Up	PRR11	2.23819316
Up	PCDHB8	2.229748135
Up	CENPI	2.224908345
Up	RPS19	2.2236319
Up	LYPD1	2.21446979
Up	GPR39	2.210783965
Up	CACNA2D4	2.2061408625
Up	FBN3	2.19616861
Up	CHST4	2.19530442

Up	GGT5	2.19431255
Up	IFITM3	2.191992825
Up	MSN	2.19076069999999
Up	SIX5	2.190561485
Up	CALD1	2.18987285
Up	EVC	2.189081575
Up	MYC	2.18898258
Up	SLC1A5	2.182315035
Up	CASZ1	2.1798857
Up	TLE6	2.17865386
Up	APCDD1L	2.177378725
Up	CD48	2.1768553775
Up	OLFML2A	2.174794845
Up	FAM111A	2.1731659
Up	TMEM154	2.172385995
Up	NT5DC2	2.17157731
Up	GBX2	2.1698997875
Up	SPRY1	2.16906218
Up	TNFRSF1A	2.167742275
Up	DPYSL3	2.1654716
Up	TNIP2	2.163364385
Up	SLC47A2	2.16178617
Up	ID4	2.157434
Up	CABP4	2.15740994
Up	SH3BP2	2.15388745
Up	S1PR2	2.15099538
Up	HMG20B	2.15037392
Up	FCGRT	2.14504555
Up	MYD88	2.141725895
Up	PSRC1	2.139027855
Up	CDCA5	2.138981215
Up	ANTXR2	2.13485667
Up	EGFLAM	2.134728435
Up	FKBP7	2.13368954
Up	RHOJ	2.132664685
Up	CISH	2.13104718
Up	LTBP1	2.1242282
Up	CD1D	2.124072695
Up	BTN3A3	2.12375769
Up	FLT4	2.123639635
Up	ZAP70	2.12115183
Up	HOXA11	2.12026302
Up	SYNE2	2.1180543
Up	ZAR1	2.109522355
Up	MICA	2.10816691

Up	FLNC	2.10696235
Up	SH2D3A	2.105060165
Up	RREB1	2.1050397
Up	CMTM3	2.104660715
Up	ITGB8	2.10026785
Up	CLECL1	2.0988293975
Up	TRIM47	2.09826555
Up	PDIA4	2.098251875
Up	STON1	2.095862695
Up	LAMA4	2.088680325
Up	MCM2	2.08808041
Up	TMCO4	2.08618046
Up	G0S2	2.085992035
Up	PHKG1	2.08465093
Up	RND3	2.082098755
Up	RAB13	2.08116014
Up	TBXA2R	2.07909438
Up	EFNA4	2.07297571
Up	ZC3HAV1	2.066279375
Up	ECM2	2.062704165
Up	LAMC1	2.055234975
Up	PPP1R14B	2.05493321
Up	PHLDA1	2.0524911
Up	ITPRIPL1	2.05142681
Up	THBS3	2.050929935
Up	GBE1	2.05045615
Up	PARP14	2.048726125
Up	KCNQ1	2.048548355
Up	PPIC	2.045516625
Up	APOBEC3B	2.0419594175
Up	BOC	2.03930328
Up	ADGRE2	2.03867103
Up	CD300A	2.038358705
Up	TCF3	2.03742302
Up	PDLIM4	2.036369505
Up	LIMD1	2.03010667
Up	POFUT1	2.028024025
Up	STK32B	2.02605244
Up	HOXD12	2.02387066
Up	EFEMP2	2.02328335
Up	CASP6	2.01790071
Up	PCDHB11	2.01764375
Up	DENND2A	2.015998225
Up	BCL2L12	2.014853755
Up	ASAP3	2.01340623

Up	MCM7	2.011069315
Up	STK17B	2.00921642
Up	PRKD2	2.008413365
Up	AURKA	2.00461108
Up	DRAM1	2.003882195
Up	MYO7A	2.00207259
Up	GNG5	2.00010207

Regulation	mRNA	logFC
Down	RELN	-8.56838372
Down	GRIN1	-8.298311227
Down	UNC13C	-8.0413108
Down	OPALIN	-7.944347485
Down	GABRA1	-7.89202856
Down	MOBP	-7.85160668
Down	RASGRF1	-7.756797675
Down	SLC17A7	-7.75317665
Down	TRHDE	-7.69649696
Down	GABRB2	-7.55847555
Down	KCNJ3	-7.49036963
Down	KSR2	-7.455946395
Down	HCN1	-7.36340519
Down	NEFM	-7.33677261
Down	MAG	-7.27412557
Down	GABRG2	-7.268722525
Down	SPOCK3	-7.25506684
Down	SV2B	-7.20935677
Down	PCLO	-7.147340235
Down	VSNL1	-7.1261632
Down	SYT4	-7.10760172
Down	FSTL5	-7.02178966
Down	PAK5	-7.014026075
Down	PACSIN1	-6.985024165
Down	CDH18	-6.980432295
Down	GRM4	-6.97813627
Down	RYR2	-6.97280534
Down	WSCD2	-6.89336112
Down	FAM153B	-6.819291836
Down	GABRG1	-6.79233569
Down	MAL2	-6.790164755
Down	SH3GL3	-6.75285732
Down	SYNPR	-6.750816084
Down	SH3GL2	-6.72712169
Down	OLFM3	-6.718117082
Down	CSMD3	-6.69771395

Down	SYT13	-6.6809363
Down	FRMPD4	-6.634365795
Down	HS6ST3	-6.63325505
Down	SNAP25	-6.62773357
Down	SLITRK4	-6.624826565
Down	GRM1	-6.623890185
Down	SYT1	-6.60510932
Down	AMER3	-6.58639141
Down	ATP8A2	-6.57787884
Down	TMEM130	-6.57763123
Down	LGI3	-6.57274616
Down	SULT4A1	-6.566261465
Down	RIMS2	-6.55950396
Down	SRRM4	-6.55368283
Down	CACNA1B	-6.51467584
Down	CKMT1A	-6.51448954
Down	RIMS1	-6.49476809
Down	SNAP91	-6.47662542
Down	NDST3	-6.445071165
Down	GAD2	-6.404822635
Down	HRH3	-6.393667325
Down	AFF2	-6.39126272
Down	ATP2B3	-6.36718723
Down	CDH7	-6.3598857
Down	SLC6A15	-6.31602882
Down	ASIC2	-6.283390465
Down	SVOP	-6.245636045
Down	RBFOX1	-6.22834926
Down	NEFL	-6.223437905
Down	NKAIN2	-6.184106545
Down	EPHA6	-6.17490993
Down	CBLN1	-6.168931325
Down	SLC8A2	-6.167068695
Down	FAM153A	-6.165414785
Down	SLC12A5	-6.16402957
Down	GABRB3	-6.13975725
Down	KCNJ12	-6.112829314
Down	UNC5D	-6.1074281
Down	CNDP1	-6.103907475
Down	CKMT1B	-6.09474205
Down	KCNK1	-6.08205499
Down	CABP1	-6.07375333
Down	GRIN2A	-6.06872925
Down	MBP	-6.05336234
Down	FA2H	-6.04316731

Down	CNTN4	-6.031765201
Down	CALY	-6.02709673
Down	CNNM1	-5.976886574
Down	SLC6A7	-5.970121535
Down	GRM5	-5.96902228
Down	GJB1	-5.96767474
Down	LRFN5	-5.955929995
Down	INA	-5.94367114
Down	PPP2R2C	-5.93620657
Down	GJB6	-5.889320023
Down	CDH12	-5.88277383
Down	RASAL1	-5.858114055
Down	LRP2	-5.852418175
Down	CDR1	-5.8466141
Down	SPHKAP	-5.83759462
Down	SEC14L5	-5.83372976
Down	EGR4	-5.83319006
Down	PTPRR	-5.830611515
Down	KCNC2	-5.81385906
Down	CNTNAP4	-5.80927019
Down	CELF4	-5.80489173
Down	TMEM132D	-5.79722618
Down	CLVS2	-5.784240715
Down	SLITRK1	-5.78367958
Down	MTUS2	-5.77988478
Down	ANKRD18A	-5.758760596
Down	SNCB	-5.75686284
Down	SLC6A17	-5.745620975
Down	PTGDS	-5.743849085
Down	VSTM2B	-5.73749719
Down	NPTX1	-5.72935557
Down	NEUROD6	-5.727186405
Down	MYT1L	-5.72386238
Down	KRT222	-5.721225594
Down	NOS1	-5.69379848
Down	TMEM125	-5.673903694
Down	CNTN3	-5.67208895
Down	CALN1	-5.66620568
Down	CCKBR	-5.656246085
Down	HOOK1	-5.65086346
Down	ELAVL2	-5.63939012
Down	SLC35F3	-5.63309735
Down	CA10	-5.632805735
Down	CNTNAP2	-5.630398705
Down	EPB41L4B	-5.62797223

Down	AJAP1	-5.62401491
Down	CHD5	-5.62126796
Down	TMEM151A	-5.620166645
Down	RBFOX3	-5.619906415
Down	C1QL3	-5.616281825
Down	DLGAP3	-5.61534013
Down	CPNE6	-5.60965162
Down	PLCH2	-5.60631828
Down	PTPN5	-5.594036325
Down	AK5	-5.579445685
Down	SLC1A6	-5.576989585
Down	MAP7D2	-5.57570097
Down	CACNG2	-5.573591909
Down	PEX5L	-5.572239
Down	PDE1A	-5.56307088
Down	HMGCLL1	-5.555135257
Down	STXBP6	-5.55064304
Down	GRIN2B	-5.54340797
Down	SFRP1	-5.53989039
Down	PHYHIP	-5.53513897
Down	CCSER1	-5.53428842
Down	CACNG3	-5.50262793
Down	CUX2	-5.490740979
Down	CAMK4	-5.48538633
Down	MPP7	-5.482686683
Down	FRRS1L	-5.478468395
Down	COL19A1	-5.477456195
Down	GALNT9	-5.476979755
Down	TMEM235	-5.47226285
Down	CPLX2	-5.46092881
Down	SCRT1	-5.45649864
Down	RTN4RL1	-5.44973738
Down	SAMD12	-5.44103628
Down	HPCAL4	-5.43364263
Down	RALYL	-5.417025095
Down	GABBR2	-5.39742383
Down	NEUROD2	-5.39628837
Down	VWC2	-5.39096659
Down	IQSEC3	-5.38712161
Down	MAL	-5.378452705
Down	CNTN5	-5.374212705
Down	ZNF385B	-5.372276005
Down	ANKRD18B	-5.370925582
Down	ERMN	-5.367848
Down	FAM163B	-5.366410125

Down	GRM3	-5.354754068
Down	DLGAP2	-5.34950485
Down	PPP1R16B	-5.34812804
Down	C11orf87	-5.33010323
Down	RAB3C	-5.32404043
Down	LRRC7	-5.32281448
Down	WNK2	-5.30418011
Down	PABPC1L2B	-5.303890369
Down	NYAP2	-5.30333077
Down	GABRA2	-5.298808735
Down	PLP1	-5.28528275
Down	CHGB	-5.281805035
Down	GALNTL6	-5.271164794
Down	PCDH11X	-5.268373765
Down	UGT8	-5.263650385
Down	GPR27	-5.257392225
Down	NRIP3	-5.249222165
Down	EPHA10	-5.240248105
Down	FSTL4	-5.223105685
Down	KCNA1	-5.22216249
Down	HTR5A	-5.221234695
Down	NWD2	-5.20379611
Down	MCF2	-5.201102664
Down	COL26A1	-5.195765395
Down	HTR2A	-5.178214375
Down	BHLHE22	-5.17564624
Down	RGS7	-5.16063995
Down	TMEFF2	-5.160168165
Down	GALNT13	-5.160039825
Down	SHANK2	-5.15711272
Down	RTN4R	-5.150749312
Down	TUBB4A	-5.14995281
Down	STMN2	-5.140309895
Down	GABRD	-5.13776533
Down	CDH8	-5.120947305
Down	PCDH7	-5.11898425
Down	CES4A	-5.11078214
Down	SLIT3	-5.109876655
Down	ACBD7	-5.107205575
Down	SNCG	-5.094154575
Down	MFSD4A	-5.09148127
Down	MAP3K9	-5.07795059
Down	CDK5R2	-5.07680606
Down	SV2C	-5.076294675
Down	ARHGAP44	-5.0744431

Down	NRG3	-5.07223354
Down	GLT1D1	-5.067650351
Down	PENK	-5.063763835
Down	ACVR1C	-5.06358749
Down	ERC2	-5.057836285
Down	DOK6	-5.04792752
Down	PRKCG	-5.03755414
Down	SYN1	-5.02984646
Down	C4orf50	-5.025840375
Down	RSPO2	-5.022837522
Down	RCAN2	-5.018398055
Down	CCDC177	-5.016226402
Down	ABCG4	-5.01087616
Down	PABPC1L2A	-5.00664311
Down	NAP1L2	-4.99921503
Down	GREM1	-4.99519954
Down	HECW1	-4.98417861
Down	ZNF536	-4.964110065
Down	KIAA0319	-4.9623658
Down	S1PR5	-4.95882144
Down	GABRG3	-4.954131105
Down	CD22	-4.94723926
Down	STXBP5L	-4.94611638
Down	SCN2B	-4.930832545
Down	HAPLN2	-4.92978626
Down	PPP4R4	-4.928953845
Down	LRTM2	-4.924310707
Down	GABRA4	-4.92198727
Down	GPC5	-4.919910685
Down	MKX	-4.917072134
Down	CACNA2D3	-4.914368885
Down	XKR7	-4.908878405
Down	CDKL2	-4.90728331
Down	DYNC1I1	-4.9029276
Down	KLHL32	-4.901552845
Down	CRYM	-4.891424537
Down	PHF24	-4.88917914
Down	SYT9	-4.88473743
Down	CDH22	-4.880060155
Down	GPR12	-4.879027305
Down	CLEC2L	-4.874857134
Down	LGR5	-4.874616735
Down	ACTL6B	-4.866112005
Down	BCAS1	-4.86460374
Down	CRTAC1	-4.860022545

Down	OPCML	-4.85589627
Down	PNMA3	-4.84984806
Down	KCNA4	-4.849029392
Down	SNCA	-4.844659675
Down	RGS7BP	-4.8430409
Down	TPPP	-4.840859
Down	DRD1	-4.84077396
Down	SHANK1	-4.83882868
Down	PTPN3	-4.829808815
Down	SLC32A1	-4.82201031
Down	ADARB2	-4.807516715
Down	SLC7A14	-4.79986675
Down	C1QTNF4	-4.798211885
Down	TMEM151B	-4.79396108
Down	PRKG2	-4.78893795
Down	NELL1	-4.783521645
Down	PRSS3	-4.783054705
Down	VIPR1	-4.77858442
Down	ZDHHC11B	-4.76661666
Down	CAMK1G	-4.764092195
Down	SGCZ	-4.7571676
Down	ANKRD34C	-4.749961925
Down	SOHLH1	-4.748284635
Down	SIAH3	-4.742499175
Down	MACROD2	-4.71880477
Down	KCNK9	-4.717690145
Down	KCNB2	-4.71424656
Down	MDGA2	-4.71418162
Down	GPR61	-4.70303
Down	CADM3	-4.7017851
Down	PRMT8	-4.697196605
Down	TCERG1L	-4.694334955
Down	KCTD16	-4.69144003
Down	FADS6	-4.68963956
Down	TUNAR	-4.683938519
Down	RAB3A	-4.68092627
Down	EDIL3	-4.67759572
Down	ENPP5	-4.67689824
Down	FGF9	-4.673051685
Down	CPNE9	-4.671143225
Down	CACNA1E	-4.66745876
Down	KCNT1	-4.66190879
Down	CACNA1I	-4.65508616
Down	ST8SIA3	-4.65275424
Down	L1CAM	-4.64254509

Down	CHRNA4	-4.632336975
Down	SLC24A2	-4.629819725
Down	SOWAHA	-4.61667685
Down	PCDH11Y	-4.615412655
Down	RNF43	-4.614203455
Down	ATP1A3	-4.61222827
Down	SYN2	-4.61216821
Down	ENPP2	-4.60950689
Down	REPS2	-4.6025107
Down	LDB3	-4.592441835
Down	TF	-4.589108185
Down	PRKCZ	-4.57781764
Down	NPM2	-4.5755169
Down	BEX5	-4.57379431
Down	PRKCB	-4.565416145
Down	ATP2B2	-4.55590712
Down	ANK3	-4.55198961
Down	IL1RAPL1	-4.540701125
Down	PTPRT	-4.537313345
Down	RANBP17	-4.531929945
Down	SPTB	-4.52918022
Down	CELF5	-4.52395071
Down	SYN3	-4.521074258
Down	JPH3	-4.5167005
Down	LCNL1	-4.503801635
Down	EPHB6	-4.50171074
Down	TCTE1	-4.499997525
Down	PVALB	-4.4989324
Down	HS3ST4	-4.498362385
Down	ST18	-4.495889145
Down	KLK6	-4.48518168
Down	ZNF488	-4.482865975
Down	NDN	-4.479735325
Down	RAB11FIP4	-4.47058291
Down	MGAT4C	-4.46874495
Down	RPH3A	-4.465308255
Down	PHACTR3	-4.464971555
Down	SSTR2	-4.46278637
Down		
Down	JAKMIP1	-4.45785374
Down	GPR83	-4.453766705
Down	RPS6KA6	-4.45042464
Down	TRPM6	-4.448778945
Down	NKX6-2	-4.448758555
Down	SERPINI1	-4.443342885

Down	CARNS1	-4.44277815
Down	PAIP2B	-4.44183516
Down	SLC9A2	-4.4394694
Down	SLC7A10	-4.439456642
Down	PTPRN	-4.43548203
Down	MIPOL1	-4.433464035
Down	SGSM1	-4.43270604
Down	ARHGDIG	-4.42859611
Down	SYNGR3	-4.424553625
Down	SLC5A11	-4.42284663
Down	HLF	-4.39878733
Down	DGKE	-4.397817465
Down	CHRNA2	-4.39780472
Down	ANKS1B	-4.39532395
Down	TRIM67	-4.38958368
Down	SSTR1	-4.38518743
Down	SLC4A10	-4.38386831
Down	FGF5	-4.377865345
Down	PRLHR	-4.375928825
Down	ANKRD30BL	-4.37249427
Down	PSD	-4.37225614
Down	DOC2B	-4.3610261
Down	CDH19	-4.35604531
Down	NEFH	-4.350007245
Down	BRINP1	-4.34964177
Down	UNC5A	-4.340347635
Down	CLDN11	-4.33655728
Down	CPLX1	-4.329159135
Down	ANO4	-4.32276369
Down	SYP	-4.31811115
Down	CACNA2D1	-4.31607107
Down	RAB3B	-4.31446522
Down	SCN3B	-4.29042375
Down	SRCIN1	-4.28677557
Down	MATK	-4.28527407
Down	PPFIA2	-4.28457751
Down	KHDRBS2	-4.282183445
Down	PRRG3	-4.276901175
Down	NALCN	-4.2754436
Down	CNTN2	-4.27272308
Down	GRM2	-4.269293913
Down	MYH14	-4.26628194
Down	SLITRK5	-4.26542881
Down	ASPA	-4.25519145
Down	HPCA	-4.25245382

Down	OPRM1	-4.25211643
Down	PLPP2	-4.2387499
Down	JAKMIP3	-4.23852775
Down	CHRM2	-4.229136465
Down	VWA5B2	-4.228906325
Down	AIFM3	-4.22638635
Down	RIPPLY2	-4.22511765
Down	FGF12	-4.222969855
Down	NMNAT2	-4.21489516
Down	XK	-4.21481261
Down	GRIP1	-4.21332836
Down	HHATL	-4.21275101
Down	CYP4X1	-4.199299
Down	TRPC5	-4.194860975
Down	NEURL1	-4.18754403
Down	SSTR3	-4.183304935
Down	RUNDC3A	-4.16666338
Down	CAMSAP3	-4.160245145
Down	ARPP21	-4.15178864
Down	SRRM3	-4.14637969
Down	ZCCHC12	-4.14605963
Down	CPXM2	-4.137733787
Down	PLCXD3	-4.132655695
Down	EXTL1	-4.121840065
Down	USH1C	-4.121627537
Down	ACP7	-4.118475675
Down	OGDHL	-4.11743112
Down	IPCEF1	-4.116049465
Down	ENPP4	-4.10863828
Down	KCNJ9	-4.10659508
Down	NEGR1	-4.10475083
Down	CDS1	-4.102917545
Down	ADCYAP1	-4.100926895
Down	KCNJ6	-4.09936333
Down	KLHL1	-4.098424825
Down	TRPV6	-4.09345239
Down	RIT2	-4.091268548
Down	FLT3	-4.084223545
Down	POU6F2	-4.079870431
Down	PDIA2	-4.0749104
Down	RIMS3	-4.06595969
Down	ELOVL7	-4.0617612
Down	SYT7	-4.059586415
Down	IQCJ	-4.057326166
Down	SPX	-4.056730152

Down	TUBA4A	-4.054926455
Down	KIF6	-4.053783615
Down	CORO6	-4.046987185
Down	CLCA4	-4.046190665
Down	PKP2	-4.042573785
Down	SCN2A	-4.035830165
Down	SYCE1	-4.03341667
Down	HTR2C	-4.02909905
Down	B4GALT6	-4.02833185
Down	TUSC3	-4.023109175
Down	SHISA8	-4.022330644
Down	CALB2	-4.019423445
Down	SOWAHB	-4.014095045
Down	GNG3	-4.00708848
Down	KCNH1	-4.00345684
Down	FBXO2	-4.002635265
Down	CNGB1	-4.002096775
Down	ISLR2	-4.00096596
Down	GJD2	-3.998197725
Down	NECAB1	-3.99488408
Down	GSTM5	-3.99357831
Down	TTC9B	-3.984798375
Down	HS3ST5	-3.97798775
Down	TMEM88B	-3.972399675
Down	ADCY1	-3.96988921
Down	CELF3	-3.969023785
Down	MYRF	-3.96603899
Down	SLC22A6	-3.95614125
Down	SMIM10L2B	-3.95163778
Down	CAMK2B	-3.94749673
Down	NHLH2	-3.94192408
Down	BICDL1	-3.93698111
Down	PYGM	-3.9283428
Down	FUT9	-3.9175961
Down	WNT7B	-3.90723559
Down	AATK	-3.8959424
Down	BEND4	-3.89005751
Down	PANX2	-3.88666691
Down	OPRK1	-3.88625494
Down	HHIP	-3.883708875
Down	ADAM11	-3.88245607
Down	GLB1L3	-3.882357875
Down	BASP1	-3.8790875
Down	PTPN20	-3.87527092
Down	DNAJC6	-3.86467919

Down	CACNA1A	-3.86062821
Down	CNTN6	-3.85470553
Down	HSPA12A	-3.85356974
Down	PLCL1	-3.84893071
Down	ERBB4	-3.84877486
Down	KIF12	-3.8405873
Down	JPH4	-3.83761466
Down	PCP4	-3.836753001
Down	ADAP1	-3.8363942
Down	CORO2A	-3.835183815
Down	OLFM1	-3.83495132
Down	NAV3	-3.83155692
Down	STXBP1	-3.8306693
Down	STX1B	-3.81276821
Down	ANXA3	-3.812739215
Down	PPP1R1A	-3.80958958
Down	GSTO2	-3.808278862
Down	DLGAP1	-3.80631478
Down	EIF4E1B	-3.80589155
Down	SLC25A48	-3.800028882
Down	SOX10	-3.797573655
Down	UBE2QL1	-3.796309025
Down	TAGLN3	-3.79272988
Down	HIPK4	-3.79181058
Down	SMIM22	-3.791167827
Down	PLD5	-3.78081907
Down	RPRML	-3.77994741
Down	DIRAS2	-3.77231203
Down	FAM71C	-3.771886994
Down	NRG1	-3.76930974
Down	FAIM2	-3.76751301
Down	SH3TC2	-3.764529105
Down	TDRD6	-3.76198602
Down	MTMR7	-3.756107745
Down	TMEM229A	-3.754736905
Down	PRR18	-3.752895365
Down	HPSE2	-3.746477665
Down	MAGEE1	-3.741759385
Down	AGAP2	-3.73557997
Down	SHISA7	-3.730894955
Down	ERBB3	-3.72788399
Down	ANKRD34A	-3.72301321
Down	FAM131C	-3.7222415286
Down	FGF17	-3.72027756
Down	KCNK12	-3.71625749

Down	RSPO3	-3.704848275
Down	PITPNM3	-3.70416926
Down	GOLGA7B	-3.698541885
Down	ACSL6	-3.69694187
Down	KRTAP5-2	-3.68494626
Down	RFPL1	-3.681723135
Down	FOLH1	-3.67744587
Down	CPLX3	-3.67114825
Down	KCNH3	-3.66805201
Down	CLGN	-3.665019283
Down	LCN12	-3.659777735
Down	FUT1	-3.656364445
Down	KIRREL3	-3.656188775
Down	GREM2	-3.64659049
Down	ASPHD1	-3.645581535
Down	PCP4L1	-3.641581965
Down	FBXL16	-3.62445299
Down	SYT12	-3.613791225
Down	AMPH	-3.611495985
Down	CAMKK1	-3.60962593
Down	ANO5	-3.60929664
Down	ZNF208	-3.60108334
Down	ADRA1B	-3.59358564
Down	EEF1A2	-3.58768918
Down	CA7	-3.585367589
Down	PPM1H	-3.57711586
Down	ALDH1A1	-3.576744385
Down	PNMA6A	-3.576011265
Down	ANK1	-3.57195039
Down	APOD	-3.57160305
Down	RBM11	-3.56622086
Down	TUBA4B	-3.5592444
Down	NRSN1	-3.55322142
Down	DOCK3	-3.548731
Down	ATP8A1	-3.54593874
Down	DMTN	-3.54292059
Down	PIP5K1B	-3.5401383
Down	SYT10	-3.537342125
Down	DUSP26	-3.534970935
Down	SLC7A4	-3.532885835
Down	LRFN2	-3.530274875
Down	CHRM4	-3.52859002
Down	FAM155B	-3.52723829
Down	TMCC2	-3.52698973
Down	KCNB1	-3.526804505

Down	MCTP1	-3.52368411
Down	TSPYL5	-3.52222239
Down	ZNF365	-3.518176835
Down	HCN2	-3.51623942
Down	SEZ6L2	-3.51538367
Down	ANKRD29	-3.5112637
Down	ZC3H12B	-3.51108469
Down	CAPN13	-3.506167435
Down	KCNA2	-3.50586516
Down	DNAJC5G	-3.505661234
Down	RIMKLA	-3.50329434
Down	CARMIL2	-3.50080012
Down	SFTPC	-3.49695575
Down	RUNDC3B	-3.49529486
Down	TMEM35A	-3.49429357
Down	FAM133A	-3.486442
Down	SLC13A5	-3.48087028
Down	NKAIN1	-3.480521995
Down	DOC2A	-3.48023247
Down	TMEM144	-3.47896522
Down	CALB1	-3.47396466
Down	CADPS2	-3.47234873
Down	RNF212	-3.471557412
Down	ADRB1	-3.469539555
Down	GFOD1	-3.46869127
Down	CNTNAP5	-3.46588715
Down	NRXN3	-3.46394167
Down	PABPC5	-3.462287395
Down	DLG2	-3.461544195
Down	CA11	-3.45472425
Down	HTR1A	-3.45380956
Down	PSG4	-3.452988675
Down	KLK7	-3.447165225
Down	RIIAD1	-3.439070685
Down	NTSR2	-3.428135222
Down	FAM95C	-3.427885357
Down	CYP2J2	-3.42396322
Down	UNC79	-3.4188296
Down	LOXHD1	-3.41660479
Down	ITIH2	-3.41608003
Down	CACNA2D2	-3.41300312
Down	CCBE1	-3.408440929
Down	KIAA0513	-3.40728486
Down	SGIP1	-3.40329433
Down	NCR3LG1	-3.402550095

Down	MYH15	-3.39861792
Down	RASGEF1C	-3.39681714
Down	NGB	-3.395042765
Down	FAM155A	-3.394156275
Down	SPTBN2	-3.39352434
Down	PBOV1	-3.392874605
Down	ATP1B1	-3.39212542
Down	STMN4	-3.38982394
Down	INPP5J	-3.387071325
Down	DNM3	-3.38553989
Down	CA4	-3.38488984
Down	MMD2	-3.375124255
Down	STEAP2	-3.37412768
Down	OTUD7A	-3.36660727
Down	CPEB3	-3.35801157
Down	RTBDN	-3.34533958
Down	ABCC8	-3.34034998
Down	EPHA8	-3.3363528
Down	KCNC3	-3.325033815
Down	GALNT14	-3.32435991
Down	STRC	-3.32020958
Down	PPP1R14A	-3.31923214
Down	TMEM179	-3.31300413
Down	PDE10A	-3.31213405
Down	DISP2	-3.311414905
Down	SVIP	-3.307809485
Down	SLC22A15	-3.30316136
Down	KNDC1	-3.30037729
Down	RAP1GAP2	-3.29813204
Down	F5	-3.297846715
Down	NAP1L3	-3.29576696
Down	NECTIN1	-3.294655975
Down	KCNJ11	-3.29362836
Down	OPN4	-3.2846323
Down	RAB37	-3.28217509
Down	CNTN1	-3.27785587
Down	SYT3	-3.276574265
Down	BSN	-3.27579485
Down	FBLL1	-3.27127263
Down	CRHR1	-3.271077345
Down	CBFA2T3	-3.266377375
Down	PLEKHD1	-3.25974124
Down	MCF2L2	-3.255479045
Down	KIF5A	-3.25421695
Down	NAPB	-3.24761835

Down	NXPH2	-3.231226594
Down	NPPC	-3.228249435
Down	GOT1	-3.22749207
Down	SYT2	-3.21962732
Down	CLEC3B	-3.218775925
Down	CAMK2N2	-3.214946535
Down	TCEAL6	-3.213092725
Down	NPFFR1	-3.21085364
Down	KLHL3	-3.20258925
Down	SYNGR1	-3.20100872
Down	KCNH8	-3.1988742
Down	PLA2G7	-3.195004775
Down	SUSD5	-3.19267686
Down	KCNK3	-3.191412065
Down	BTBD8	-3.18735074
Down	ALOXE3	-3.187080344
Down	CABLES1	-3.17836015
Down	SCN1B	-3.17805427
Down	HBA1	-3.17522319
Down	PCSK6	-3.168993695
Down	POPDC3	-3.168900344
Down	PDE11A	-3.16346204
Down	FLG	-3.16279517
Down	RAB33A	-3.16068287
Down	CHRNB2	-3.156923245
Down	KCNIP2	-3.156720675
Down	GPR17	-3.154526805
Down	FAXC	-3.15414957
Down	PLCXD2	-3.15259524
Down	BEGAIN	-3.151465
Down	KIF1A	-3.151016375
Down	RAB40B	-3.14709494
Down	SDR16C5	-3.1470149
Down	GRIN2C	-3.144818995
Down	RAI2	-3.144173405
Down	TMEM63C	-3.143702045
Down	SEMA3G	-3.14145917
Down	NCDN	-3.13922159
Down	PAK6	-3.13579044
Down	CDK5R1	-3.1334187
Down	FBXO27	-3.124375245
Down	KIAA1549L	-3.12275053
Down	RNF207	-3.117254155
Down	CLCN4	-3.11639414
Down	IL34	-3.112193645

Down	PNPLA5	-3.11159795
Down	USP44	-3.107772435
Down	RAPGEF5	-3.10754189
Down	PDZD7	-3.1058432
Down	RAPGEF4	-3.103533035
Down	RFPL2	-3.096320355
Down	NPTXR	-3.094707795
Down	DAB1	-3.090383795
Down	SLC27A2	-3.088560566
Down	ZDHHC11	-3.085065035
Down	INPP5F	-3.08483664
Down	DUSP8	-3.08479487
Down	MTURN	-3.081808665
Down	CASKIN1	-3.07721205
Down	GNAI1	-3.07610028
Down	WFDC1	-3.074331315
Down	MYOT	-3.07237032
Down	LYNX1	-3.07006692
Down	ABLIM2	-3.0692297
Down	MICAL2	-3.05548252
Down	ARFGEF3	-3.05242454
Down	SLC26A8	-3.051475445
Down	SLC8A3	-3.0477759
Down	PGR	-3.0398476
Down	LINC00634	-3.025268115
Down	CNKSR1	-3.020925185
Down	OCA2	-3.01817555
Down	CLUL1	-3.0125590834
Down	ST6GALNAC1	-3.008280919
Down	KIT	-3.006113025
Down	MAPK8IP2	-3.00417302
Down	EPB41L3	-2.99813964
Down	CDKL5	-2.99466973
Down	PGBD5	-2.990546805
Down	FAAH	-2.98361409
Down	NSF	-2.98334835
Down	GPR63	-2.976877375
Down	STX1A	-2.97682187
Down	BEX2	-2.976411785
Down	UNC13A	-2.97601884
Down	SLITRK2	-2.97180354
Down	VSX1	-2.971262135
Down	APLP1	-2.96881815
Down	CPEB1	-2.962893965
Down	SHTN1	-2.959686445

Down	ARRB1	-2.94987241
Down	KIF5C	-2.946218225
Down	SCN8A	-2.94581061
Down	ANKRD62	-2.945654525
Down	SCG3	-2.94302189
Down	TSPAN7	-2.94119684
Down	CAMKK2	-2.938091615
Down	GRAMD1B	-2.93713202
Down	QDPR	-2.9334168
Down	KCNC1	-2.9300329
Down	KCNK4	-2.929825118
Down	NELL2	-2.92957315
Down	MAST1	-2.92549797
Down	BRSK2	-2.92088126
Down	PSG7	-2.9186848
Down	ABCA10	-2.91777967
Down	LRTM1	-2.917068872
Down	GAL3ST1	-2.911857795
Down	NPAP1	-2.908049762
Down	PDE1B	-2.90780717
Down	SEMA3D	-2.903833915
Down	P2RX5	-2.90304988
Down	ADAMTS8	-2.902621685
Down	GNAL	-2.90079872
Down	AKR1E2	-2.89422046
Down	SORBS2	-2.893104245
Down	KCNAB2	-2.887226735
Down	NINJ2	-2.88515978
Down	RXRG	-2.885093585
Down	CLVS1	-2.88326607
Down	GRIK2	-2.882369235
Down	ST6GALNAC3	-2.87419697
Down	RASGRF2	-2.87379225
Down	CACNB4	-2.86989329
Down	SELENBP1	-2.865751955
Down	CYP4Z1	-2.863782375
Down	SLC6A20	-2.859835605
Down	AQP7	-2.85573067
Down	GPR158	-2.85568442
Down	ZNF483	-2.85517257
Down	ANXA8L1	-2.8519964575
Down	DMRT2	-2.851187175
Down	FAM189A1	-2.843053467
Down	ELOVL4	-2.841561785
Down	RAD21L1	-2.837806303

Down	STXBP5	-2.83268794
Down	FBXO41	-2.83059919
Down	EIF4E3	-2.829611635
Down	GJC2	-2.82796927
Down	HTR3B	-2.8233083
Down	BUB1B-PAK6	-2.82090553
Down	NRXN1	-2.81902505
Down	CLSTN3	-2.81482898
Down	PSG1	-2.806162993
Down	CDYL2	-2.80510707
Down	PDXP	-2.80473891
Down	SCAMP5	-2.80366095
Down	CMTM5	-2.803511885
Down	PSG9	-2.80329586
Down	CEND1	-2.80283935
Down	ZNF727	-2.799092845
Down	AMIGO1	-2.79776686
Down	ZBTB18	-2.7976209
Down	MAP7	-2.79620984
Down	IGSF21	-2.792884995
Down	DNAJA4	-2.79065096
Down	MALRD1	-2.7863121
Down	NT5DC1	-2.781974555
Down	UNC80	-2.77545295
Down	BDNF	-2.77489234
Down	TGFBR3L	-2.767877765
Down	MYRIP	-2.76503546
Down	PANK1	-2.76119254
Down	NDRG4	-2.7611047
Down	KCNA5	-2.759325899
Down	FCHO1	-2.75832465
Down	ABCC12	-2.7558474575
Down	FXYD1	-2.752472855
Down	SLC45A3	-2.75168072
Down	SPAG6	-2.74991798
Down	SPINT2	-2.74896553
Down	ADCY5	-2.74369719
Down	PLEKHH1	-2.74112902
Down	RS1	-2.73637184
Down	KLHL34	-2.723060295
Down	IGLON5	-2.7175019
Down	KCNQ2	-2.71377926
Down	PPFIA3	-2.71305983
Down	ACOT7	-2.711771725
Down	NOX5	-2.704901925

Down	CAMTA1	-2.70434256
Down	TEF	-2.69706112
Down	MFSD6	-2.69645951
Down	MICU3	-2.6899107
Down	UTS2	-2.689782735
Down	RERG	-2.68866177
Down	ENHO	-2.684913585
Down	CYFIP2	-2.68070655
Down	HBD	-2.6804428
Down	INSM2	-2.680386265
Down	CATSPER2	-2.67840845
Down	SIDT1	-2.676024885
Down	MAST3	-2.6751753
Down	SEMA6B	-2.672424
Down	RBP3	-2.66903549
Down	SGPP2	-2.664041255
Down	CACNA1D	-2.662335485
Down	AGBL1	-2.661896145
Down	RPS6KA5	-2.65396465
Down	PLCB1	-2.65037198
Down	NCOA7	-2.64669455
Down	MYO1D	-2.644066875
Down	ATL1	-2.64259512
Down	ZNF728	-2.637845794
Down	AGTPBP1	-2.63451472
Down	RHBDL1	-2.631220665
Down	HID1	-2.63010887
Down	KCNA3	-2.630002535
Down	FNDC5	-2.62749283
Down	DSCAML1	-2.624552975
Down	PAK1	-2.615876825
Down	RAB26	-2.614915045
Down	CLMN	-2.61185884
Down	MGAT3	-2.61054764
Down	B4GALNT1	-2.61022047
Down	DNM1	-2.60697795
Down	C10orf90	-2.60641097
Down	ADGRA1	-2.60221927
Down	DCAF12L2	-2.5973800625
Down	RTN1	-2.59599247
Down	IGSF8	-2.59518357
Down	SHANK3	-2.588890685
Down	AMER2	-2.58558239
Down	C14orf132	-2.58556861
Down	ASCL5	-2.583133984

Down	AP3B2	-2.58293279
Down	TACR3	-2.582165169
Down	CDH10	-2.58048745
Down	C10orf95	-2.57782687
Down	SLC6A1	-2.5770163
Down	HRNR	-2.57506589
Down	CYP2E1	-2.57254929
Down	TUBA8	-2.571346925
Down	VAMP2	-2.56870518
Down	DOCK9	-2.56151548
Down	PSG6	-2.55676523
Down	BOK	-2.554402445
Down	MOAP1	-2.54064094
Down	TBC1D30	-2.537243235
Down	C16orf92	-2.537111249
Down	CHADL	-2.536737895
Down	GARNL3	-2.53205277
Down	DNAJC12	-2.531271005
Down	EFHD1	-2.53096175
Down	EDN3	-2.530220837
Down	CACNA1S	-2.530163335
Down	GPR62	-2.52675556
Down	IL5RA	-2.5229173075
Down	RERGL	-2.522118585
Down	PSG8	-2.52152107
Down	GPR6	-2.51960595
Down	THEM5	-2.513227155
Down	NEBL	-2.51228465
Down	NHLRC1	-2.51040153
Down	EFCAB5	-2.50991575
Down	SMIM10L2A	-2.504938145
Down	MYOM1	-2.50151169
Down	ATCAY	-2.501055445
Down	ADGRB3	-2.500649075
Down	DBNDD2	-2.49994327
Down	EPCAM	-2.499003475
Down	ERICH3	-2.49565626
Down	PPP3CB	-2.4937994
Down	PARD6A	-2.487956225
Down	RHOV	-2.484214245
Down	C21orf91	-2.48243672
Down	LPAR3	-2.4821265365
Down	PRKCE	-2.481612435
Down	GPRASP1	-2.48123681
Down	SEMA4D	-2.47933365

Down	MAP1A	-2.473317475
Down	MMP17	-2.472740575
Down	LCN15	-2.4725624175
Down	NAP1L5	-2.471372415
Down	CNKSR2	-2.47065207
Down	EHD3	-2.47056558
Down	PIP4K2A	-2.465396055
Down	GPR85	-2.4643217
Down	PHACTR1	-2.46139852
Down	MPL	-2.46018947
Down	RYR3	-2.45958429
Down	TSPYL1	-2.45813634
Down	ALDOC	-2.457432115
Down	TMEM266	-2.45728972
Down	CD55	-2.456413405
Down	S100A1	-2.455292005
Down	ABCA5	-2.44654456
Down	RNF165	-2.443607445
Down	PCDHAC2	-2.44300725
Down	DNAH2	-2.440323325
Down	THPO	-2.437924384
Down	LY6K	-2.437748637
Down	FAM171A1	-2.431668345
Down	CADM2	-2.4233813
Down	BEX1	-2.422868455
Down	GDAP1L1	-2.4202776
Down	SLC1A1	-2.412886055
Down	PRPH2	-2.410410805
Down	TSPOAP1	-2.4102803
Down	LPCAT4	-2.40975134
Down	WASF1	-2.405471125
Down	PKP4	-2.4049503
Down	GPR143	-2.404405572
Down	GNAO1	-2.401504475
Down	CASQ2	-2.39856864
Down	LIN28B	-2.398488175
Down	PCDH15	-2.396460095
Down	NRG4	-2.39031725
Down	CAMK1D	-2.38207762
Down	LNX1	-2.38002727
Down	C9orf24	-2.379091895
Down	PLCH1	-2.376276745
Down	ROGDI	-2.37493757
Down	LRRC4	-2.37270765
Down	SV2A	-2.36925578

Down	TMEFF1	-2.368013715
Down	ABCA2	-2.366335125
Down	KCNIP4	-2.36093857
Down	PSG3	-2.359934
Down	PRKAR1B	-2.35816987
Down	BFSP1	-2.356694645
Down	GNB5	-2.356504075
Down	LHFPL5	-2.35580472
Down	MGMT	-2.35164592
Down	MLXIPL	-2.350678115
Down	MYO5A	-2.35055575
Down	BAMBI	-2.350373485
Down	CLEC4G	-2.349163435
Down	PATE4	-2.347346895
Down	EBF3	-2.346788857
Down	FRMPD1	-2.34589998
Down	RPS6KL1	-2.345790235
Down	ADRA2B	-2.339806667
Down	LZTS3	-2.33172689
Down	SYNJ2	-2.32874091
Down	SYNDIG1L	-2.32655976
Down	YPEL2	-2.32569356
Down	NIPAL3	-2.32511249
Down	TACR2	-2.32491333
Down	CGREF1	-2.322976795
Down	ATOH7	-2.32052053
Down	NUAK1	-2.31572255
Down	CTXN2	-2.313329603
Down	ACY3	-2.311496322
Down	RBP7	-2.310395258
Down	PER3	-2.30885405
Down	CALM3	-2.30650195
Down	IDS	-2.3054049
Down	RPP25	-2.30536878
Down	NPTN	-2.3043319
Down	FABP6	-2.30381845
Down	SLC24A4	-2.303413115
Down	HBQ1	-2.301211616
Down	NCS1	-2.30105775
Down	MAP6D1	-2.29803066
Down	IGFL4	-2.29600106
Down	GABARAPL1	-2.29550621
Down	ESYT3	-2.294446715
Down	CALHM1	-2.29279926
Down	OMG	-2.289186745

Down	GLRB	-2.285028445
Down	HR	-2.283900645
Down	EPB41L1	-2.28146145
Down	CAP2	-2.280829195
Down	ACADSB	-2.27950483
Down	CACNA1C	-2.276778075
Down	PRKAR2B	-2.276229215
Down	RAPGEFL1	-2.27497277
Down	SMPD3	-2.27408699
Down	PLCL2	-2.269888545
Down	SPTBN4	-2.26886431
Down	PRR36	-2.267340765
Down	SEMA4F	-2.26257007
Down	PAQR8	-2.26242542
Down	PELI3	-2.258365125
Down	LARP6	-2.258296945
Down	SNPH	-2.25612038
Down	ENTPD3	-2.251429085
Down	PNLDC1	-2.246985904
Down	SERPINA10	-2.2453113525
Down	CCDC184	-2.241943435
Down	ZDHHC22	-2.24185442
Down	CAMK2G	-2.24064917
Down	LAMA3	-2.23998321
Down	FAM171A2	-2.237541805
Down	LYPD6B	-2.237232975
Down	NIPA1	-2.23694331
Down	TSPYL2	-2.23584107
Down	SNURF	-2.23342242
Down	PREPL	-2.22851885
Down	CEP126	-2.22834144
Down	APBB1	-2.22791732
Down	ADAMTSL3	-2.225113815
Down	FAM171B	-2.2246597
Down	LANCL1	-2.22440369
Down	HOMER1	-2.22398407
Down	FAM124A	-2.222576105
Down	SULT1A1	-2.21995489
Down	SH3BGRL2	-2.21972031
Down	TMEM59L	-2.21948196
Down	LHFPL1	-2.218652102
Down	SLC6A12	-2.214767095
Down	PPP1R1B	-2.21463343
Down	ALDOB	-2.211986595
Down	RIMS4	-2.20921095

Down	GRK3	-2.208951815
Down	CYB561D2	-2.199834775
Down	CACNB2	-2.19383099
Down	STK32C	-2.19328252
Down	BICDL2	-2.193235915
Down	SLC25A27	-2.19316584
Down	CNP	-2.1913558
Down	PSG11	-2.187415229
Down	ST8SIA6	-2.187351647
Down	STARD4	-2.185260045
Down	FBXW7	-2.17867577
Down	TNR	-2.17731267
Down	TNFSF9	-2.177178035
Down	MSANTD3-TMEFF1	-2.17624448
Down	RAB6B	-2.1747899
Down	PRLR	-2.17416586
Down	NBEA	-2.173577175
Down	NTM	-2.1668512
Down	C17orf107	-2.161721745
Down	PLG	-2.16063462
Down	PDE3B	-2.157915095
Down	R3HDM1	-2.15670767
Down	LONRF2	-2.1478592
Down	RELL2	-2.14776668
Down	ADAM22	-2.14483653
Down	SSTR4	-2.14271265
Down	TBC1D9	-2.142124735
Down	IQSEC2	-2.13947363
Down	CBX7	-2.13919824
Down	OXR1	-2.1391219
Down	HDAC11	-2.13761392
Down	HECW2	-2.13704262
Down	TTPA	-2.136867515
Down	CACNB3	-2.136505555
Down	LSM11	-2.13630443
Down	CHRD	-2.131366985
Down	PKIA	-2.13125497
Down	CERCAM	-2.13031741
Down	CEP170B	-2.130254365
Down	PPP3R1	-2.12962603
Down	RASGRP1	-2.123573515
Down	PPP3CA	-2.12310310000001
Down	ELAVL3	-2.12252513
Down	C3orf49	-2.121264965
Down	CALM1	-2.119939675

Down	PPIP5K1	-2.11784209
Down	TNFRSF25	-2.11476969
Down	PRNP	-2.11085765
Down	SPRN	-2.10986399
Down	CD200	-2.1084895
Down	OPRL1	-2.108419745
Down	FAM234B	-2.10706478
Down	PRRT2	-2.105078285
Down	HPRT1	-2.10116811
Down	SH3BP5	-2.10051961
Down	PPFIA4	-2.0998495
Down	RTN3	-2.09638332500001
Down	PTGES3L	-2.09466339
Down	TEK	-2.09104963
Down	PSD3	-2.0896114
Down	TCEAL5	-2.08838673
Down	LRRC73	-2.087431335
Down	BSCL2	-2.08733519
Down	LYPD5	-2.08431683
Down	PPM1L	-2.08237405
Down	TNNI3K	-2.082078365
Down	WDR37	-2.07570805
Down	RNF112	-2.07412372
Down	GDPD1	-2.07342984
Down	RAB15	-2.07247836
Down	BEX4	-2.07212465
Down	ASRGL1	-2.071904965
Down	OR2AK2	-2.07154955
Down	SYS1-DBNDD2	-2.069598195
Down	SSBP3	-2.06561475
Down	UNC5C	-2.059576915
Down	PTPN4	-2.059068255
Down	MAPK10	-2.0580661
Down	SEC61A2	-2.055982905
Down	BDH1	-2.055670315
Down	FAM13C	-2.050951945
Down	CACNA1F	-2.042516255
Down	NXPE3	-2.0386777
Down	CYP11A1	-2.0347681725
Down	SCAI	-2.03456144
Down	MAPRE2	-2.03355355
Down	INPP5A	-2.03245887
Down	REEP1	-2.02636003
Down	C14orf180	-2.023866765
Down	TPRG1L	-2.02354589

Down	SLC3A1	-2.019688845
Down	KIF3A	-2.01873138
Down	SSX2IP	-2.01802961
Down	LHPP	-2.015639835
Down	BSPRY	-2.015096187
Down	ECE2	-2.01475618
Down	SLC9A6	-2.01437101
Down	MRLN	-2.014050475
Down	PSG5	-2.01090514
Down	NAT8L	-2.008174
Down	RAP1GAP	-2.00513375
Down	MTMR8	-2.003201232
