# Supplementary material for: Identification of Deregulated miRNAs and mRNAs Involved in Tumorigenesis and Detection of Glioblastoma Patients Applying Next-Generation RNA Sequencing
Source: Pharmaceuticals (Basel). 2025 Mar 19;18(3):431. doi: 10.3390/ph18030431 (PMC11944724; doi:10.3390/ph18030431)
Supplement: Supplementary file 1 [file pharmaceuticals-18-00431-s001.zip › Table S3. The list of primer sequences used for validation by RT-qPCR.pdf]

Table S3. The list of primer pairs used for RT-qPCR measurements.

	**Forward primer (5′-3′)**	**Reverse primer (5′-3′)**
MYBL2	CGGAGCAGAGGGATAGCAAG	TCCAGTCCTGCTGTCCAAAC
AURKB	ACCCCATCTGCACTTGTCCT	TCGGGTGTCCCACTGCTATT
VEGFA	CTACCTCCACCATGCCAAGT	GATAGACATCCATGAACTTCACCA
CDC45	CGGATGCTGCACAACCATTT	GTGCGTCCAGAAACTTGCTC
E2F2	GAAGGCCAAGAACAACATCCAG	TTGTCCTCAGTCAGGTGCTT
HOXC10	CACCTCGGATAACGAAGCGA	GGGCACCTCTTCTTCCTTCC
HOXD13	AGTCCTGGACGCTGGCTA	CCCTCTTCGGTAGACGCAC
HRH3	CTGCTCAACCTCGCCATCTC	CTGTCAGCACGTAGGGTACA
CBLN1	AGAACGCAGCACTTTCATCG	CCAGCGAAGGCTGAAATCAC
RELN	ATACAGCGTCAACAACGGGA	CGACCTCCACATGGTCCAAA
HCN1	AGACAGTTGCCATTGACCGA	CTGCACCATCTCCCTGTCAT
NEUROD6	CAGGAGACGATGCGACACT	TTTGCTTCTGGTCCTCGCA
PRLHR	TTCTTCCCGCGAGTGCTTT	CTCTGGTTGGCGGGAGTTG
FABP6	TCACCGGCAAGTTCGAGATG	TGCGGGCCTTTTCGATTACA
GAPDH2	GTCTCCTCTGACTTCAACAGCG	ACCACCCTGTTGCTGTAGCCAA
